# Identification and validation of biomarkers related to nicotinamide metabolic pathway activity in heart failure

**DOI:** 10.3389/fgene.2025.1673314

**Published:** 2025-12-16

**Authors:** Jianping Du, Xiaoyu Yang, Shuxing Wu, Shuli Bi, Peng Wang, Lisong Cheng, Zhuhua Yao

**Affiliations:** 1 Department of Cardiology, Tianjin Union Medical Center, The First Affiliated Hospital of Nankai University, Tianjin, China; 2 School of Medicine, Nankai University, Tianjin, China

**Keywords:** NDC1, NUP133, heart failure, metabolism-related, TRMT11

## Abstract

**Background:**

Heart failure (HF) is the final stage of cardiovascular diseases. Nicotinamide metabolism (NMN) plays a key role in cardiovascular dysfunction. We aimed to explore genes correlated with NM pathway activity (NMRGs) in HF.

**Methods:**

HF data were obtained from public databases, and NMRGs were sourced from literature. Weighted gene co-expression network analysis (WGCNA) identified NM-associated module genes. Candidate genes were selected via differential expression profiling and module analysis. Biomarkers were identified using protein-protein interaction (PPI) networks, machine learning, and gene expression validation. Diagnostic efficacy was assessed via nomogram. Functional enrichment, immune infiltration, and drug prediction analyses were performed. Biomarker expression was validated by Reverse Transcription quantitative Polymerase Chain Reaction (RT-qPCR).

**Results:**

Among 492 candidate genes, NDC1, NUP133, and TRMT11 were validated as biomarkers. The nomogram showed high diagnostic accuracy. Biomarkers were enriched in spliceosome and ubiquitin-mediated proteolysis pathways. Immune infiltration revealed correlations with neutrophils. Potential drugs, including tetradecanoylphorbol acetate, were identified. Biomarker expression was significantly lower in HF.

**Conclusion:**

NDC1, NUP133, and TRMT11 are NM-related biomarkers in HF, offering insights into HF pathogenesis and therapy.

## 1 Introduction

Heart failure (HF), the final stage in the progression of various cardiovascular diseases (CVDs), is characterized by abnormal ventricular filling or ejection function resulting from a range of structural or functional cardiac abnormalities (McDonagh et al., 2021). HF is a condition that significantly diminishes quality of life and inflicts substantial social and financial burden, and therefore has become a primary focus of clinical prevention and treatment efforts (Mamas et al., 2017). The causes of HF include ischemic heart disease, cardiomyopathy, valvular heart disease, hypertension, diabetes, arrhythmias, systemic toxins, and cardiotoxic drugs (Kemp and Conte, 2012). According to the 2021 statistical update from the American Heart Association, the incidence and prevalence rates of HF continue to rise (Deng et al., 2024). The 5-year mortality rate of HF remains approximately 50%, indicating a poor prognosis (Roger, 2021). The exploration of the pathogenesis and treatment methods for HF has been ongoing, evolving from the traditional “Golden Triangle“ (A combination of angiotensin-converting enzyme inhibitors/angiotensin receptor blockers, beta-blockers, and mineralocorticoid receptor antagonists) to the “New Four Links“ (The combination therapy regimen of angiotensin-converting enzyme inhibitors (ACEI)/angiotensin II receptor blockers (ARB)/angiotensin receptor-neprilysin inhibitors (ARNI), beta-blockers, mineralocorticoid receptor antagonists (MRA), and sodium-glucose cotransporter 2 inhibitors (SGLT2i)), and now to the contemporary “Five Golden Flowers” (On the basis of ‘New Four-Links,’ soluble guanylate cyclase (sGC) is added, forming a combination therapy regimen with five core drugs.) treatment era (Authors/Task Force et al., 2024). However, the prognosis for HF remains poor, and further exploration and understanding of its pathogenesis are necessary.

Nicotinamide adenine dinucleotide (NAD^+^) is a crucial coenzyme in cellular energy metabolism, playing a significant role in catabolic reactions and oxidative phosphorylation processes. Meanwhile, NAD^+^ serves as a substrate for various NAD^+^-dependent enzymes that regulate key cellular metabolic and signaling pathways (Abdellatif et al., 2021; Chase and Rieling, 1986). In HF, myocardial stress significantly affects the expression of the rate-limiting enzyme nicotinamide phosphoribosyltransferase (NAMPT) in the NAD^+^ salvage pathway. In addition, a decrease in NAD^+^ levels and alterations in the NAD^+^/NADH redox status have been observed in cardiac metabolic diseases of various etiologies (Ju et al., 2023). Preclinical studies have demonstrated that aimed at reducing NAD^+^ consumption or promoting NAD^+^ production can enhance intracellular NAD^+^ levels or normalize NAD^+^/NADH redox states, thereby exhibiting cardioprotective effects across multiple models (Wu Y. et al., 2024). However, despite the significant potential demonstrated by these interventions, the mechanisms underlying the benefits of increasing NAD^+^ levels remain not fully understood, and translating this therapy into clinical practice continues to encounter numerous challenges. Nicotinamide Mononucleotide (NMN), as a precursor of NAD^+^, is implicated in the development of cardiovascular dysfunction and diseases (Chase and Rieling, 1986). Some clinical studies have preliminarily confirmed the effectiveness and safety of NMN supplementation, indicating its potential role in cardiovascular protection, and no significant adverse reactions have been observed (Wang et al., 2021). The relationship between HF and NMN is primarily evident in how fluctuations in NAD^+^ levels affect cardiac function. Furthermore, there is potential for treating HF by modulating NAD^+^ metabolism. Therefore, a comprehensive exploration of the relationship between HF and NM is anticipated to yield new strategies and targets for the treatment of HF.

This study identified differentially expressed genes correlated with NM pathway activity (NMRGs) in HF based on transcriptome data from public databases. Additionally, biomarkers were obtained through machine learning and expression validation. Enrichment analysis and immune infiltration analysis were conducted on these biomarkers, providing new insights for the in-depth exploration of the mechanisms of action of NMN in HF and its clinical treatment.

## 2 Materials and methods

### 2.1 Data collection

Data on HF were retrieved from the GEO database (https://www.ncbi.nlm.nih.gov/geo/). The GSE59867 dataset (Sequencing platform: GPL6244) was employed as a training set, which included 34 HF peripheral blood mononuclear cell (PBMC) and 30 control PBMC samples. The GSE66360 dataset (Sequencing platform: GPL570) served as a validation set, which comprised 49 HF whole blood samples and 50 control whole blood samples. Furthermore, a comprehensive review of the literature yielded a total of 42 genes associated with NMRGs (Supplementary Table S1) (Cui et al., 2023).

### 2.2 WGCNA

In order to assess the statistical significance of the differences in NMRGs between HF and control groups, NMRGs were evaluated across all samples within the training set utilizing the ssGSEA algorithm from the “GSVA” (v 1.42.0) (Hanzelmann et al., 2013). The NMRGs scores were then analyzed for differences between the 2 groups using the Wilcoxon test (P < 0.05).

To construct a weighted gene co-expression network on all samples from the training set, the “WGCNA” (v 1.18.0) (Langfelder and Horvath, 2008) was used. Hierarchical clustering analysis was performed for all samples using the Euclidean distance metric to identify and exclude outliers in expression profiles. In order to determine an appropriate soft threshold, the pickSoftThreshold function was utilized to calculate the average number of connections and the *R* ^2^ (scale-free fitting index) for various soft thresholds. Additionally, the pickSoftThreshold function was employed to evaluate alternative soft thresholds. According to the hybrid dynamic tree cutting algorithm, the genes were divided into modules with a minimum gene number of 50 and a merging parameter of 0.25. Spearman correlation analysis was carried out by using the cor function, and the correlation between the modules and the NMRGs scores was evaluated with the criterion of P < 0.05 and |cor|> 0.3, so as to identify the modules with the strongest correlation as the key modules; finally, the key module genes were filtered out by setting thresholds for the membership of the modules (MM > 0.6) and gene significance (GS > 0.3) thresholds were set to screen out key module genes.

### 2.3 Differential expression analysis

Based on the training set, the “limma” software package (v3.54.0) (Love et al., 2014) was used to analyze the differences between the heart failure group (HF group) and the control group to obtain differential genes, and the genes related to the differential expression of HF patients were identified (adj.P < 0.05). Moreover, the “ggplot2” (v 3.4.1) (Gustavsson et al., 2022) was utilized to construct a volcano plot of DEGs, and the top 10 genes (by |log2FC| from high to low) with significant up- and downregulation differences were labeled, and then the heatmap of these genes was drawn using the “pheatmap” (v 1.0.12) (Wu C. et al., 2024).

### 2.4 Identification of candidate genes and functional enrichment

To identify the genes associated with NMN in HF, the “VennDiagram” (v 1.7.3) (Chen et al., 2011) was employed to determine the intersection of DEGs and module genes. These genes were subsequently documented as candidate genes for further analysis. Using the “clusterProfiler” package (v 4.2.2) (Yu et al., 2012), Gene Ontology (GO) and KEGG pathway analyses were carried out candidate genes to identify enriched biological functions and pathways (adjusted P < 0.05).

### 2.5 PPI network, machine learning, and expression validation

The candidate genes were delivered to the STRING database (https://string-db.org/) with a confidence score set at 0.4. The PPI network was downloaded, and Cytoscape software (v 3.8.2) (Shannon et al., 2003) was utilized to identify core sub-network within the network. Candidate core genes linked to this sub-network were identified based on the scores were selected for subsequent analyses.

We used the least absolute shrinkage and selection operator (LASSO) to analyze the training set to improve the accuracy of candidate core gene selection. The “glmnet” (v 4.1.8) (Friedman et al., 2010) was employed to perform LASSO regression of candidate core genes. The optimal lambda value was identified using 10-fold cross-validation to screen for candidate biomarkers.

A study was instituted to evaluate the expression levels of potential biomarkers in HF compared to control groups, utilizing the GSE59867 and GSE66360 datasets. The Wilcoxon test was implemented the analysis of these datasets (P < 0.05). Genes that demonstrated consistent expression patterns and significant differences between the groups across both datasets were identified as candidate biomarkers.

### 2.6 Developing and assessing a nomogram

Within the confines of the training set, a nomogram model was formulated, underpinned by biomarkers, utilising the “rms” (v 6.5.0) in order to determine the likelihood of the occurrence of HF. Construct a calibration curve and determine the accuracy of the nomogram by “rms.” Meanwhile, the clinical impact curve (CIC) was drawn by the “clinicalrisk” (v 4.1.0) (Luo et al., 2023) to evaluate the potential application of the nomogram in clinical practice.

### 2.7 Gene set enrichment analysis (GSEA) and gene set variation analysis (GSVA)

GSEA was carried out with the objective of elucidating the biological functions of biomarkers throughout the progression of HF. The initial step in the research was to calculate the Spearman correlation coefficients between the various biomarkers and every single gene across the entire range of samples from the training set, with the “psych” (v 2.1.6) (Robles-Jimenez et al., 2021) being utilized for the purpose. Following that, the sorted genes were then arranged in descending order according to their respective Spearman correlation coefficients, with this ranking then being utilized as the gene set that was tested in further analyses. Meanwhile, the reference gene set (c2.cp.kegg.v7.0.symbols.gmt) was retrieved from the MSigDB (https://www.gsea-msigdb.org/gsea/msigdb). Subsequently, the GSEA was performed employing the “clusterProfiler,” with a threshold of adj. P < 0.05.

The target gene sets for GSVA analysis were obtained from the MsigDB database. The ssGSEA algorithm in ‘GSVA’ was utilized to assess the GSVA scores of each gene set in different samples. Subsequently, the differences in scores between the HF and control groups were compared by Wilcoxon test (P < 0.05, |t| > 2).

### 2.8 Chromosome localization, functional similarity, and GeneMANIA network of biomarkers

In this study, the “RCircos” (v 1.2.2) (Zhang et al., 2013) was referred to determine the chromosome localization of biomarkers. To further analyze the functional similarity among biomarkers, the GO semantic similarity of these genes was calculated using the “GOSemSim” (v 2.26.1) (Zhang et al., 2013) (P > 0.5). In an effort to make a more in-depth prediction of the interactions between biomarkers and their involved biological functions, the building of a gene co-expression network was carried out by the GeneMANIA website (http://www.genemania.org/).

### 2.9 Immune infiltration analysis

The relative infiltrating cell populations of 22 immune cells (Yang et al., 2021) were quantified by the CIBERSORT algorithm between the HF and control groups. The differences in immune cells between the two groups were compared using the Wilcoxon test (P < 0.05), and the correlations between immune cells and their interactions with biomarkers were analyzed by psych software (|r| > 0.3, P < 0.05).

### 2.10 Regulatory network construction and potential drug prediction

To investigate the molecular regulatory mechanisms of the biomarkers, 3 regulatory networks were constructed. First, the NetworkAnalyst database (https://www.networkanalyst.ca/) was utilized to predict transcription factors (TFs) targeting the biomarkers. TF-mRNA regulatory network was illustrated by Cytoscape software. In addition, microRNAs (miRNAs) targeting the biomarkers were predicted using the miRWalk database (http://mirwalk.umm.uni-heidelberg.de/) and the miRDB database (https://mirdb.org/). Key miRNAs were identified by overlapping predictions from both databases. Subsequently, a miRNA-mRNA regulatory network was constructed. Functional enrichment analysis of key miRNAs was conducted using FunRich, a tool designed for functional enrichment analysis and visualization of the biological pathways associated with enriched miRNAs. Long non-coding RNAs (lncRNAs) corresponding to each key miRNA were predicted by StarBase database (http://starbase.sysu.edu.cn/index.php). Based on the aforementioned biomarkers, key miRNAs, and lncRNAs, the lncRNA-miRNA-mRNA network was built using the Cytoscape software.

Furthermore, CTD (https://ctdbase.org/) was used to predict drugs targeting biomarkers, and visual drug-biomarker networks were constructed by Cytoscape software.

### 2.11 RT-qPCR

The study has been approved by the ethics committee or institutional review board (IRB) of Tianjin Union Medical Center. The approval number and date of approval are as follows: (2025) Expedited Trial No. (C57). For this research, blood samples were procured from 5 HF patients and 5 control samples at Tianjin People’s Hospital. The clinical information of the patient was presented in Supplementary Table S2. These specimens were then utilized for RT-qPCR analysis. The research received ethical approval from (2025) Expedited Trial No. (C57). Primer sequences employed for qPCR are presented in Supplementary Table S3. The quantitative PCR was conducted on a CFX Connect Real-Time Fluorescence Quantitative PCR Instrument (Bio-Rad, United States). Relative quantification of key genes was calculated by 2^−ΔΔCT^. The RT-qPCR data were transferred to Excel for initial organization and then statistically analyzed and graphically presented using GraphPad Prism 10 software (P < 0.05).

### 2.12 Statistical analysis

Statistical analyses were performed utilizing R software (v 4.2.2). The Wilcoxon test was employed to evaluate differences between 2 groups (P < 0.05). In the case of RT-qPCR analysis, inter-group comparisons were executed by means of the t-test (P < 0.05).

## 3 Results

### 3.1 The 2,482 module genes were associated with NMN

**FIGURE 1:**
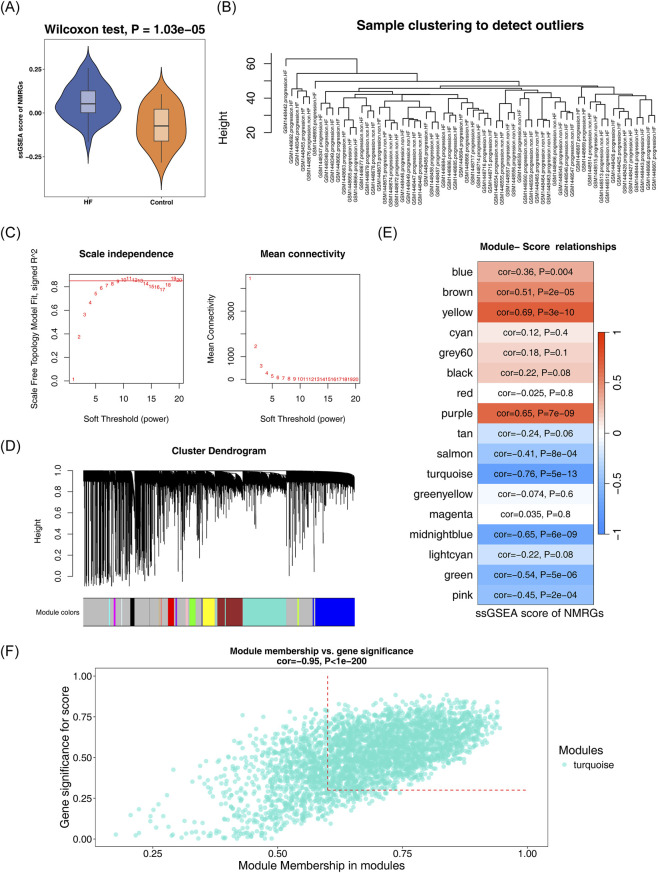
Identification of NM-associated gene modules in HF. **(A)** The violin graph shows the scores of NM-RGs related genes in HF and control groups, with significantly higher scores in HF than in control groups. **(B)** The clustering diagram shows the clustering of samples. The samples are clustered according to the size of the differences between them, and the height of the line connecting two samples is the similarity degree between them. **(C)** Scale-free topology analysis for soft-threshold selection in WGCNA. The left figure shows the scale-free fitting index at each power value, with the red line at 0.85 indicating the threshold. The first power value above this threshold should be selected. The right figure illustrates the average number of connections at each power value. The first power value where the curve flattens after connecting the average number of connections at different power values should be chosen. Considering all factors, power = 10 is selected. **(D)** Hierarchical clustering dendrogram of co-expression modules. **(E)** Module-trait relationships showing correlation with NMRG scores. Each row in the figure represents a gene module, and the correlation between nicotinamide metabolism pathway and gene module is identified by color. **(F)** Heatmap of gene expression in the key turquoise module. The scatter diagram shows the correlation score of related genes in the module and nicotinamide metabolism pathway.

The ssGSEA score of NMRGs was higher in HF group than control group (Figure 1A). In the WGCNA analysis, the results indicated that no obvious outliers were present in the samples (Figure 1B). In the process of building the scale-free network, the ideal soft threshold was identified as 10, corresponding to a scale-free *R* ^2^ of 0.85, and the mean connectivity approached but did not reach 0 (Figure 1C). Based on hierarchical cluster analysis, 17 gene modules were identified in total (Figure 1D). Subsequently, based on the correlation analysis between the modules and the ssGSEA scores, the turquoise module (r = −0.76, P < 0.0001) was identified as the key module associated with NMRGs, which contained a total of 3,972 genes (Figure 1E). By applying filtering thresholds for module membership (MM > 0.6) and gene significance (GS > 0.3), a total of 2,482 key module genes were obtained from the turquoise module for subsequent analysis (Figure 1F).

### 3.2 The 492 candidate genes were associated with NMN in HF

**FIGURE 2:**
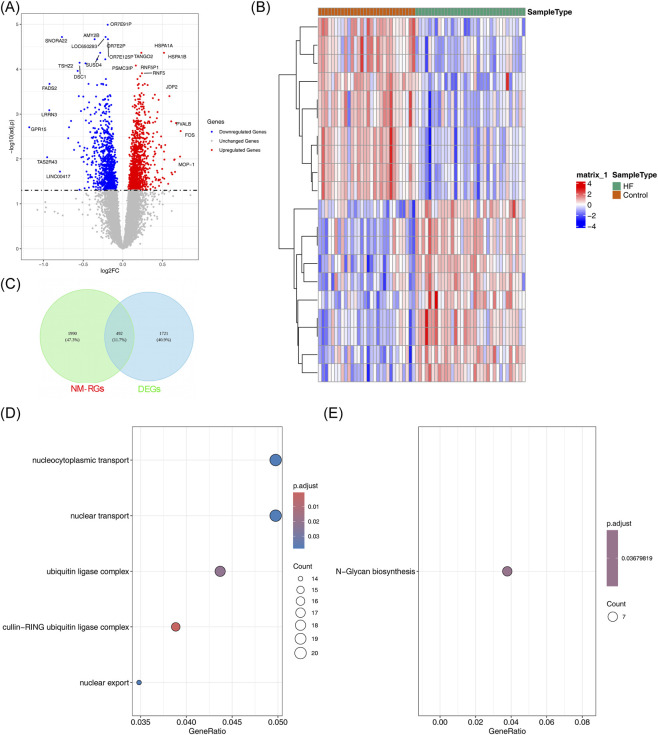
Screening of NM-related candidate genes in HF. **(A)** Volcano plot displaying DEGs between HF and control groups. The x-axis of the graph represents the ratio of gene expression values between patient and normal samples, with log2 applied. The y-axis shows the significance level of the gene expression difference, with-log10 applied. Red-marked genes are those that show significantly upregulated expression in the patient sample, while blue-marked genes show significantly downregulated expression. The top 10 genes with the most significant differences are highlighted on the graph. **(B)** Heatmap of top differentially expressed genes. Gene expression is represented by a red and blue gradient. Each row in the figure represents a gene and each column represents a sample. **(C)** Venn diagram showing intersection between DEGs and WGCNA module genes. **(D)** GO enrichment analysis of candidate genes. **(E)** KEGG pathway analysis of candidate genes. Each bubble in the figure represents an enrichment pathway, the color of the bubble represents the degree of significance, and the size of the bubble represents the number of genes enriched.

Differential expression analysis showed that there were 2,213 DEGs between 2 groups. Among them, 1,183 genes in the HF group were identified as upregulated genes, and 1,030 genes were identified as downregulated genes (Figures 2A,B). The DEGs obtained from the training set were intersected with the genes related to nicotinamide metabolism, resulting in 492 candidate genes (Figure 2C). The enrichment analysis of the 492 candidate genes disclosed associations with 9 GO terms (Supplementary Table S4). These terms encompassed a wide range of functions such as nucleocytoplasmic transport, nuclear transport, and cullin-RING ubiquitin ligase complex among others (Figure 2D). These processes are closely related to the pathogenesis of heart failure. Impaired nucleo-cytoplasmic transport can disrupt the expression of cardiac protective genes and stress response factors, as demonstrated by alterations in the nuclear pore complex in failing hearts (Tarazón et al., 2012). Meanwhile, dysfunction of the ubiquitin-proteasome system (cullin-RING lase is a key regulatory factor) leads to abnormal protein quality control and remodeling observed in HF (Nishida et al., 2015; Morreale and Walden, 2016). Additionally, KEGG pathway analysis identified only one significantly enriched pathway, ‘N-Glycan biosynthesis’ (Figure 2E). This pathway is responsible for the biosynthesis of N-linked glycans, a crucial post-translational modification for the proper folding, stability, and function of numerous membrane and secreted proteins.

### 3.3 The NDC1, NUP133, and TRMT11 were identified as biomarkers

**FIGURE 3:**
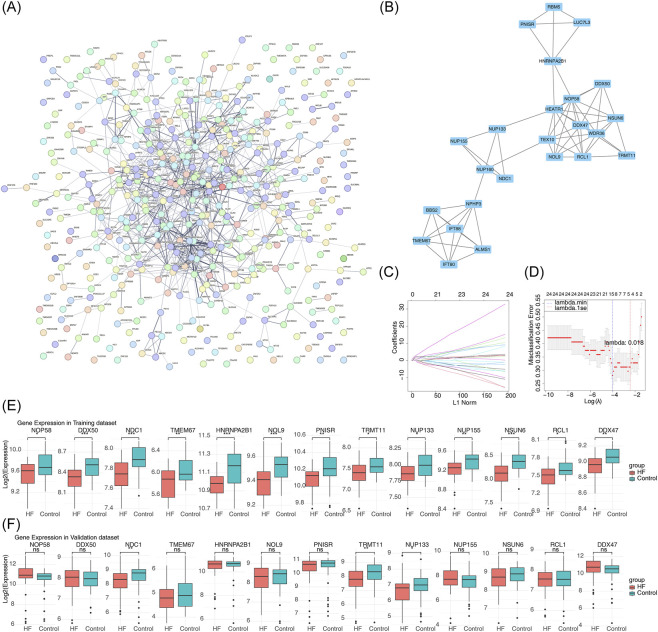
Identification and validation of NM-related biomarkers. **(A)** PPI network of candidate genes with hub nodes highlighted. **(B)** Core subnetwork extracted from PPI analysis. **(C)** LASSO coefficient profiles of candidate genes. **(D)** Cross-validation for optimal lambda selection in LASSO regression. The x-axis represents the logarithm of the penalty coefficient λ, and the y-axis shows the coefficients of each variable in the model. As λ increases, the coefficients of less important variables quickly drop to 0, while those of more important variables remain significant. The y-axis of Figure B represents the mean squared error (MSE), and the x-axis represents the value of λ (lambda.min). **(E,F)** Expression validation of candidate biomarkers in **(E)** training and **(F)** validation datasets.

In the PPI network of candidate genes, ADD3, RPL30, MRPS14, and RRM1 had frequent protein-level interactions with other genes (Figure 3A). The 24 candidate core genes in the core sub-network were further selected (Figure 3B). For result of LASSO algorithm, when the minimum lambda value was 0.018, a total of 13 candidate biomarkers were screened among the candidate core genes (Figures 3C,D). Subsequently, according to the GSE59867 and GSE66360 datasets, NDC1, NUP133, and TRMT11 exhibited markedly low expression levels in the HF group (Figures 3E,F). Consequently, 3 biomarkers—NDC1, NUP133, and TRMT11—were selected for subsequent analysis.

### 3.4 The nomogram was found to be a more effective tool for predicting HF

**FIGURE 4:**
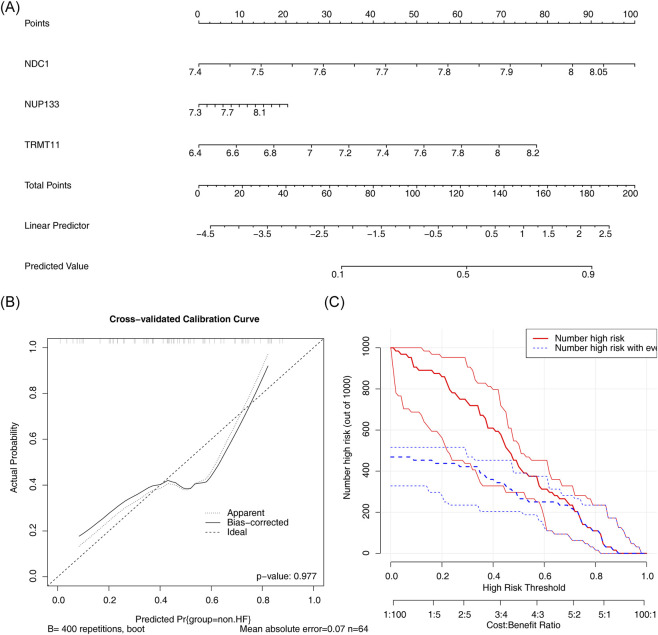
Diagnostic performance evaluation of biomarkers. **(A)** Nomogram integrating three biomarkers for HF risk prediction. **(B)** Calibration curve assessing nomogram accuracy. The horizontal axis represents the predicted probability, and the vertical axis represents the actual frequency of occurrence. The ideal calibration curve is close to the 45° line, indicating accurate prediction. **(C)** Clinical impact curve demonstrating predictive performance.

A nomogram was constructed for the purpose of predicting the risk of HF using the biomarkers (Figure 4A). It was evident from the calibration curve that there was a minimal discrepancy between the actual and predicted risks of HF (Figure 4B). In addition, CIC demonstrated a strong correlation between model predictions and actual occurrences, indicating high efficiency in clinical prediction (Figure 4C).

### 3.5 Biomarkers interactions were significantly enriched in various signaling pathways

**FIGURE 5:**
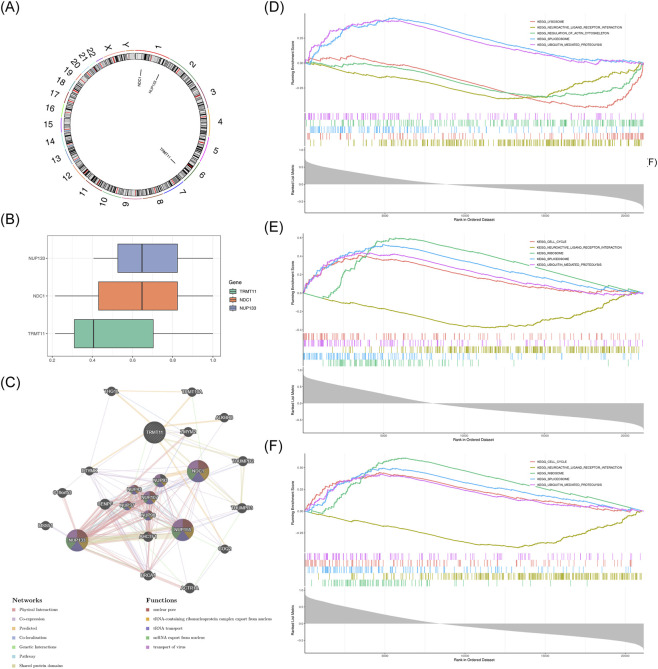
Functional characterization of NM-related biomarkers. **(A)** Chromosomal localization of three biomarkers. **(B)** Functional similarity network among biomarkers. **(C)** GeneMANIA interaction network showing functional associations. **(D–F)** GSEA enrichment plots for **(D)** NDC1, **(E)** NUP133, and **(F)** TRMT11.

NDC1 and NUP133 were distributed on chromosome 1, while TRMT11 was on chromosome 6 (Figure 5A). An exploration was conducted into the association of function between the biomarkers, and the results indicated that the functional similarity between NDC1 and NUP133 were higher (P > 0.5) (Figure 5B). In the GeneMANIA network, biomarkers interacted with 20 genes, such as NUP155, NUP93, and NUP107, and they were involved in functions such as nuclear pore, tRNA-containing ribonucleoprotein complex export from nucleus, and tRNA transport (Figure 5C). Besides, GSEA revealed that NDC1 was significantly enriched in 46 pathways, NUP133 was significantly enriched in 42 pathways, and TRMT11 was enriched in 47 pathways (Supplementary Table S5). Notably, biomarkers were co-enriched in several common pathways, such as spliceosome and ubiquitin mediated proteolysis (Figures 5D–F). The results suggested that NDC1, NUP133, and TRMT11 might contribute to cellular degradation and protein synthesis, all of which could be relevant to the pathogenesis of HF.

### 3.6 A reduction in immune cell infiltration and the activation of multiple pathways were observed in HF

**FIGURE 6:**
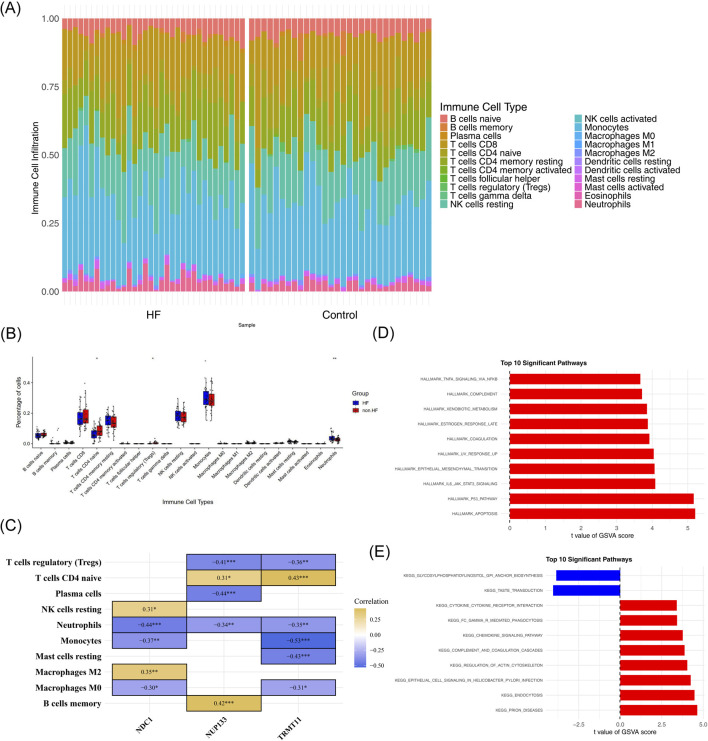
Immune infiltration and pathway analysis in HF. **(A)** Composition profile of 22 immune cell types across samples. **(B)** Differential immune cell infiltration between HF and control groups. **(C)** Correlation network between biomarkers and immune cells. **(D,E)** GSVA analysis of **(D)** Hallmark and **(E)** KEGG pathways showing differential activity between groups.

To analyze the immune microenvironment characteristics of HF, we evaluated the infiltration levels of 22 types of immune cells between the HF group and the control group using the CIBERSORT algorithm. As illustrated in Figure 6A, a graphical representation was given of the infiltration abundance percentage of each immune cell across the entire sample set. A total of 3 immune cell types exhibited significant immune cell infiltration differences between the 2 groups (Figure 6B). Of these, neutrophils significantly increased in the HF group (p < 0.05), while regulatory T cells and naive CD4 T cells significantly decreased (p < 0.05). Concurrently, regulatory T cells and naive CD4 T cells were remarkably decreased in HF group contrasted with control group. Moreover, activated CD4 memory T cells demonstrated a marked positive association with M1 macrophages (r > 0.3). And macrophages demonstrated a significant inverse association with resting natural killer (NK) cells (r < −0.3) (Figure 6C). The majority of the immune cells demonstrated a positive correlation with biomarkers (Figure 6C). Notably, NDC1 and TRMT11 exhibited the strongest correlation with neutrophils (r > 0.3).

In addition, GSVA-Hallmark analysis revealed that 10 pathways, including apoptosis, P53 pathway and IL6-JAK-STAT3 signaling, were significantly activated in the HF group (Figure 6D). GSVA-KEGG analysis revealed that 8 pathways, including prion diseases and endocytosis, exhibited significant activation in the HF group. In contrast, the pathways related to glycosylphosphatidylinositol GPI anchor biosynthesis and taste transduction demonstrated significant inhibition in the HF group (Figure 6E). The results of the study indicated that the majority of significant pathways were associated with cellular signal transduction, which might be linked to the development of HF.

### 3.7 Interaction networks were identified between biomarkers and, TFs, miRNAs, lncRNAs, and drugs

**FIGURE 7:**
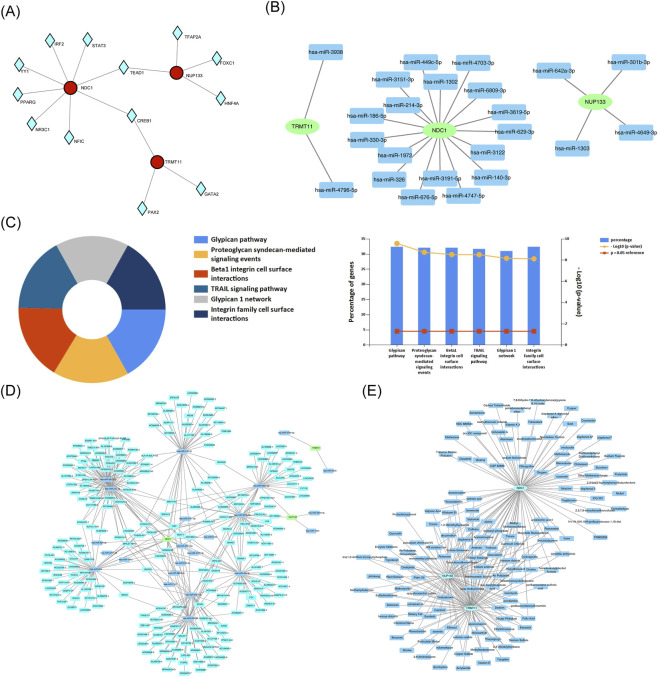
Regulatory networks and therapeutic prediction. **(A)** TF-biomarker regulatory network. **(B)** miRNA-mRNA interaction network. **(C)** Functional enrichment of key miRNAs. **(D)** lncRNA-miRNA-mRNA ceRNA network. **(E)** Drug-biomarker interaction network.

In this study, 13 TFs were identified by the prediction results from the database (Figure 7A). Specifically, 8 TFs (e.g., TEAD1, CREB1) were found to target NDC1, 5 TFs (e.g., TEAD1, CREB1) targeted NUP133, and 3 TFs (e.g., CREB1, PAX2) targeted TRMT11. In particular, CREB1 was found to regulate NDC1, NUP133, and TRMT11, while TEAD1 was identified as a co-regulator of NDC1 and NUP133. This co-targeting indicated that these TFs might have coordinated roles in regulating NDC1, NUP133, and TRMT11 expression, highlighting their potential importance in the regulatory network underlying disease pathogenesis. Additionally, some miRNAs targeting the biomarkers were identified. NDC1, NUP133, and TRMT11 were found to be modulated by 17, 4, and 2 key miRNAs, respectively (Figure 7B). The application of functional enrichment analysis revealed that the majority of key miRNAs exhibited significant enrichment in glypican pathway (Figure 7C). Furthermore, 279 lncRNAs that interacted with key miRNAs were identified through the database (Figure 7D). Among them, lncRNA (XIST) exerted a regulatory influence on NDC1 and NUP133 by regulating hsa-miR-301b-3p and hsa-miR-214-3p, respectively. These miRNAs and lncRNAs were suggested to play crucial regulatory roles in the expression of biomarkers.

Potential therapeutic interventions were explored by identifying drugs targeting CCR1, NDC1, NUP133, and TRMT11. A total of 89 drugs targeting NDC1, 71 drugs targeting NUP133, and 94 drugs targeting TRMT11 were found (Figure 7E). Tetradecanoylphorbol acetate, tetrachlorodibenzodioxin, and propylthiouracil targeting biomarkers were identified. The results of the study indicated that these drugs might have a therapeutic effect on HF by targeting biomarkers.

### 3.8 Biomarkers were significantly underexpressed in clinical samples from patients with HF

**FIGURE 8:**
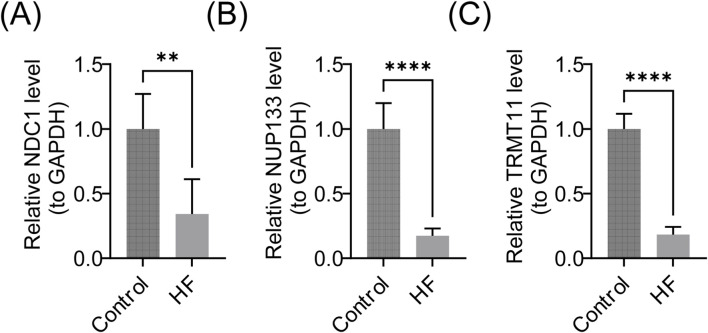
Experimental validation of biomarker expression. **(A–C)** RT-qPCR validation of **(A)** NDC1, **(B)** NUP133, and **(C)** TRMT11 expression in clinical samples from HF patients and controls.

To further validate the expression of biomarkers, we conducted RT-qPCR experiments using clinical samples. The results presented in Figures 8A–C demonstrated a significant decrease in the expression levels of NDC1, NUP133, and TRMT111 in the HF group compared to the control group (P < 0.01). These findings were consistent with the bioinformatics analyses performed, thereby providing additional support for the hypothesis that NMN significantly influenced the progression of HF.

## 4 Discussion

HF is a significant contributor to global mortality. Circulating biomarkers that reflect the pathophysiological pathways underlying the development and progression of HF may aid clinicians in the early diagnosis and management of patients with this condition (Castiglione et al., 2022; Heidenreich et al., 2022). An increasing amount of research is focusing on the intricate relationship between HF and metabolism. Among these, the relationship between NMN and HF has garnered significant attention (Wang et al., 2021). Based on the transcriptome dataset from the GEO database, this study investigated the genes involved in HF and their relationship to NMN through a series of bioinformatics analyses (Deng et al., 2024). The study analyzed the functions and immune characteristics of gene-related biomarkers using bioinformatics methods and explored their potential regulatory mechanisms.

In this study, three biomarkers associated with NMN in HF were identified and validated: NDC1, NUP133, and TRMT11. These biomarkers exhibited consistent expression trends in both the training and validation sets. NDC1 (Nuclear Division Cycle 1) is a key transmembrane nucleoporin that serves as a core component of the nuclear pore complex (NPC). It plays a central role in maintaining nuclear membrane integrity, regulating nucleocytoplasmic transport, and cell cycle progression (Mauro et al., 2022; Amm et al., 2023; Stavru et al., 2006). Previous studies have shown that in the occurrence and development of heart failure, the nuclear pore complex of cardiomyocytes undergoes significant structural and functional remodeling (Tarazón et al., 2012). In this study, we observed that NDC1 is significantly downregulated under heart failure conditions. Based on this, we hypothesize that the decreased expression of NDC1 may interfere with the normal function of the nuclear pore complex, disrupting nucleocytoplasmic transport within cardiomyocytes, thereby participating in the pathological progression of heart failure. However, the specific mechanism by which NDC1 acts in heart failure still needs to be clarified through further functional experiments.

NAs a core component of the nuclear pore complex Nup107-160 subcomplex, NUP133 plays a crucial role in maintaining nuclear membrane integrity, regulating nucleocytoplasmic transport, and ensuring the fidelity of cell mitosis (Rogg et al., 2022; Souquet et al., 2018; Bilir et al., 2019). Its functional abnormalities are closely related to the pathological processes of heart failure and various cancers. In heart failure, the structure and function of nuclear pore complexes in cardiomyocytes undergo significant changes (Tarazón et al., 2012), and persistently activated DNA damage response is a key factor driving the progression of heart failure, a process often accompanied by nuclear pore dysfunction (de Wit et al., 2023). Based on the above background, this study found that NUP133 is significantly downregulated in patients with heart failure. We hypothesize that the downregulation of NUP133 may weaken the stability of the nuclear pore complex, indirectly promoting the occurrence and development of heart failure. Therefore, the abnormal expression of NUP133 provides a potential mechanistic insight into understanding nuclear dysfunction in myocardial cells under heart failure conditions, and also offers valuable research clues for further exploration of the functions of nuclear pore-related molecules in cardiac diseases.

TRMT11 is an important RNA modification regulatory protein in eukaryotes. As a common partner of various methyltransferases, it is widely involved in the methylation process of key RNA molecules such as tRNA, rRNA, and translation termination factors, playing a core role in maintaining the function of the translation machinery (Wang et al., 2025). Heart failure, as a complex clinical syndrome, is closely related to multiple pathological mechanisms such as myocardial cell apoptosis, fibrosis, metabolic disorders, and inflammatory responses (Fonseka et al., 2025). In recent years, studies have shown that post-transcriptional regulation plays a key role in cardiac remodeling: changes in microRNA expression profiles have been systematically identified in children with heart failure and are closely associated with inflammation and myocardial injury (Ragusa et al., 2019), while epigenetic changes such as DNA methylation have also been widely reported in cardiac tissues of patients with different types of heart failure (Desiderio et al., 2024). Against this background, TRMT11-mediated RNA methylation modification may serve as one of the bridges connecting cellular metabolism with cardiac function regulation. This study preliminarily analyzed the association between TRMT11 and NMN in heart failure, adding a new perspective to the in-depth understanding of the molecular mechanisms of this disease and the exploration of potential therapeutic strategies.

NDC1, NUP133, and TRMT11 were found to be co-enriched in pathways such as spliceosome splicing and ubiquitin-mediated protein hydrolysis by GSEA. spliceosome is an RNA-protein molecular machine composed of small nuclear ribonucleoproteins (snRNPs) and a large number of auxiliary non-snRNPs (Cao et al., 2024; Badr et al., 2016). Splicers function by gradually assembling a series of distinct complexes (Wahl et al., 2015). RNA splicing has emerged as a crucial process linked to potential mechanisms underlying cardiovascular disease. Changes in splicing body composition and various cardiac-related alternative splicing (AS) factors can disrupt the normal structure and homeostasis of the heart (Nishida et al., 2015; Morreale and Walden, 2016; Zhang et al., 2018). In a previous study, it was discovered that SNRPA in ischemic cardiomyopathy (ICM) directly binds to the 3′UTR of STAT5B and promotes its substitution for polyadenylation, a process that is coordinated with alternative splicing (AS) (Zhang et al., 2023). This substitution occurs when T cell receptor signaling is active (Qiu et al., 2017), thereby increasing mRNA stability and enhancing the expression of the STAT5B protein, which is involved in T cell activation and apoptosis.

Additionally, DHX15 is an adenosine triphosphate (ATP)-dependent RNA helicase that dissociates spliceosomal complexes (Ribera et al., 2021; Maul-Newby et al., 2022). Its deficiency is associated with impaired endothelial energy metabolism, particularly changes in mitochondrial complexes, which lead to a reduction in intracellular ATP production. Studies have shown that in patients with ICM, the oxidative phosphorylation system is dysregulated, leading to the production of majority of the ATP consumed by the heart in the mitochondrial endometrium (Maul-Newby et al., 2022). The above results suggest that the spliceosome can influence HF. our analysis also predicted the enrichment of biomarkers in this pathway, suggesting that they may be involved in HF through this mechanism. However, their specific mechanisms of action require further investigation in clinical trials.

The ubiquitin-proteasome system orchestrates a pivotal biological mechanism in regulating protein homeostasis. Intracellular ubiquitination mediates the coordinated clearance of misfolded, potentially toxic proteins or nonfunctional proteins, thereby preserving protein homeostasis (Nishida et al., 2015; Morreale and Walden, 2016). Intracellular protein processing and maturation depend on post-translational modifications (PTMs), which restructure proteins, modulate their activity, and culminate in functional alterations. (Zee et al., 2010; Liddy et al., 2013). Ubiquitination is a crucial component of PTM and plays a critical role in cardiac remodeling (Fessart et al., 2014; Barac et al., 2017). The specific recognition of substrates by E3 ubiquitin ligases is central to the process of ubiquitination. The interaction between E3 ubiquitin ligases and target proteins is a critical step in the protein pathway mediated by the ubiquitin proteasome system (Morreale and Walden, 2016). For example, WWP2, a HECT-type E3 ubiquitin ligase, affects biological processes by regulating multiple substrates, including membrane protein transport, signaling, and transcription (Chen et al., 2014; Li et al., 2007). Studies have shown that WWP2 is involved in isoprenaline (ISO)-induced cardiac remodeling and that WWP2 expression levels are significantly decreased during this process.

In myocardial-specific WWP2 knockout mice, the ubiquitination level of PARP1 is decreased, while the effects of ISO-induced PARP1 activation and PARylation are enhanced, thereby exacerbating ISO-induced myocardial hypertrophy, HF, and myocardial fibrosis 66 (Zhang et al., 2020). The research findings indicate that the hydrolysis of ubiquitinated proteins plays a significant role in the development of HF. Furthermore, it can be speculated that biomarkers may influence this pathway and impact HF. The results of this study suggested that NDC1, NUP133, and TRMT11 may contribute to HF through the aforementioned enriched pathways which were involved in cellular degradation and protein synthesis, potentially linking them to the pathogenesis of HF.

In addition, this study revealed significant differences in immune cell populations between the HF group and the control group, particularly in neutrophils, regulatory T cells, and naive CD4 T cells. Furthermore, there were notable correlations among various immune cells. Three biomarkers exhibited significant positive correlations with several immune cell types, including neutrophils, resting mast cells, monocytes, and resting NK cells. Neutrophils, as a key component of the innate immune system, not only coordinate inflammatory abatement and host defense, but also promote cardiac healing by polarizing monocytes/macrophages and eliminating debris in HF after activation (Horckmans et al., 2017), and preclinical and clinical evidence suggest that the increased incidence of morning myocardial infarction may be associated with neutrophil overactivation. This indicates that neutrophil activity varies according to circadian rhythms, which may contribute to the occurrence of myocardial infarction (Schloss et al., 2016). Although an increase in neutrophils can exacerbate HF, experimental depletion of neutrophils in mice with myocardial infarction (MI) can lead to worsened cardiac function, increased fibrosis, higher mortality rates, and accelerated progression to HF. Therefore, neutrophils play a crucial role in cardiac repair following MI, which aligns with our study that demonstrates a significant increase in neutrophil levels among HF patients.

Anti-inflammatories aimed at reducing neutrophil-induced injury during acute myocardial infarction have been shown to limit acute myocardial tissue damage. However, treatment strategies designed to mitigate acute neutrophil-driven inflammation after MI must be carefully balanced, as they may interfere with the healing response and cardiac remodeling (Horckmans et al., 2017). As is well known, HF is the end stage of nearly all cardiovascular diseases. Numerous research studies have demonstrated that regulatory T cells (Treg cells) play a protective role during the early stages of heart injury. Following acute myocardial infarction, the number of Treg cells in the heart and mediastinal lymph nodes of mice increases, promoting tissue repair (Weirather et al., 2014; Tang et al., 2012). The adoptive transfer of *in vitro*-Tregs cells activated reduced cardiomyocyte apoptosis following MI and inhibited neutrophil infiltration, thereby improving fibrosis and cardiac function in mice (Xia et al., 2015). Therefore, we hypothesize that biomarkers may regulate the immune microenvironment in HF by influencing the behavior and associated functions of immune cells. More studies are needed in the future to understand these effects and their regulatory mechanisms.

In general, our research provides some new clues for the regulation of heart failure, but we must also face its existing limitations. First, the sample size of clinical samples used for RT-qPCR validation is small, which to some extent affects the statistical power and universality of the results. Second, although bioinformatics analysis has revealed the correlation between NDC1, NUP133, and TRMT11 with the activity of the nicotinamide metabolic pathway, these genes have not yet been verified through functional experiments to directly regulate NAD+ metabolism and participate in the pathogenesis of heart failure. To make up for these shortcomings, future studies will, on the basis of expanding the clinical sample size, further combine cell and animal models, and deeply explore the specific mechanisms by which these genes regulate nicotinamide metabolism and the progression of heart failure through gene intervention methods, thereby providing a more solid experimental basis for elucidating the relevant mechanisms and developing potential therapeutic strategies.

## 5 Conclusion

NDC1, NUP133, and TRMT11 were identified as novel biomarkers correlated with NM pathway activity in HF, potentially interfering with signal transduction pathways and promoting HF development. The results provide a foundation for understanding the pathogenesis of HF and improving clinical diagnosis and therapy. However, this study also has several limitations. Firstly, the specific molecular mechanisms of these biomarkers in HF have not been thoroughly investigated. Additionally, although drugs targeting these biomarkers have been proposed, their actual efficacy in clinical applications and effectiveness compared to existing therapeutic drugs still require extensive support from evidence-based medicine. Therefore, future research should focus on exploring the functions of these genes and their potential applications in therapy to further validate their clinical significance.

## Data Availability

The datasets presented in this study can be found in the Gene Expression Omnibus, (https://www.ncbi.nlm.nih.gov/gds), with accession number GSE59867 and GSE66360.
