# Supplementary material for: Identification and validation of biomarkers related to nicotinamide metabolic pathway activity in heart failure
Source: Front Genet. 2025 Dec 16;16:1673314. doi: 10.3389/fgene.2025.1673314 (PMC12748002; doi:10.3389/fgene.2025.1673314)
Supplement: Supplementary file 1 [file DataSheet1.pdf]

Supplementary Material

1 Supplementary Tables

Supplementary Table 1 List of 42 nicotinamide metabolism-related genes (NMRGs) collected from literature.

AOX1
NUDT12
BST1
PNP
CD38
QPRT
ENPP1
CYP8B1
ENPP3
NADK2
NADK
NAPRT
NADSYN1
NAXD
NAMPT
NAXE
NMNAT1

NMRK2
NMNAT2
PARP10
NMNAT3
PARP14
NMRK1
PARP16
NNMT
PARP4
NNT
PARP6
NT5C
PARP8
NT5C1A
PARP9
NT5C1B
PTGIS
NT5C2
PTGS2
NT5C3A
RNLS

NT5E
SLC22A13
NT5M
SLC5A8

Supplementary Table 2 Clinical Characteristics of the Study Subjects

Patient ID	Sex	Age (years)	Infarction Location	EF (%)	NT-proBNP (pg/mL)	Comorbidities
HF1	Male	69	Anterior wall	43	6658.30	Hypertension, Diabetes
HF2	Male	65	Inferior wall	55	4495.63	Hypertension, Hyperlipidemia
HF3	Female	77	Anterior wall	42	3115.31	Hypertension, Hyperlipidemia
HF4	Male	64	Inferior wall	51	3816.85	Hypertension
HF5	Male	73	Inferior wall	47	3212.96	Hypertension
Control1	Male	61	Inferior wall	56	305.40	Hypertension, Diabetes
Control2	Male	69	Inferior wall	56	158.55	Hypertension
Control3	Male	64	Anterior wall	56	389.12	Diabetes

Patient ID	Sex	Age (years)	Infarction Location	EF (%)	NT-proBNP (pg/mL)	Comorbidities
Control4	Male	71	Inferior wall	56	216.06	Hyperlipidemia
Control5	Male	56	Inferior wall	57	225.90	Hypertension, Hyperlipidemia

Supplementary Table 3 primer sequences of RT-qPCR.

primer	sequence
NDC1 F	CAGGTCGCGGGACATACTG
NDC1 R	CAGGCCATCACCATTCCCAT
NUP133 F	AGCAGACTGACAACCAGAGC
NUP133 R	AGCAGACTGACAACCAGAGC
TRMT11 F	AGCAGACTGACAACCAGAGC
TRMT11 R	AGCAGACTGACAACCAGAGC
GAPDH F	AGCAGACTGACAACCAGAGC
GAPDH R	AGCAGACTGACAACCAGAGC

Supplementary Table 4 Gene Ontology (GO) enrichment analysis results of 492 candidate genes associated with nicotinamide metabolism in heart failure.

ONTOLOGY	ID	Description	GeneRatio	BgRatio	pvalue	p.adjust	qvalue	geneID	Count

BP	GO:0051168	nuclear export	14/402	166/18614	1.53978773464191e-05	0.0382321829168677	0.0371069775515721	FRAT2/HNRNPA2B1/NUP160/DUSP16/THOC3/SRSF3/HNRNPA1/XPO4/MALT1/SETD2/CTDSPL2/ABCE1/NUP155/NUP133	14
BP	GO:0006913	nucleocytoplasmic transport	20/402	328/18614	3.28737600317005e-05	0.0382321829168677	0.0371069775515721	FRAT2/TMEM53/HNRNPA2B1/KPNA5/NDC1/NUP160/DUSP16/THOC3/FBXO22/SRSF3/HNRNPA1/XPO4/MALT1/SETD2/CTDSPL2/IPO11/ABCE1/UBR5/NUP155/NUP133	20
BP	GO:0051169	nuclear transport	20/402	328/18614	3.28737600317005e-05	0.0382321829168677	0.0371069775515721	FRAT2/TMEM53/HNRNPA2B1/KPNA5/NDC1/NUP160/DUSP16/THOC3/FBXO22/SRSF3/HNRNPA1/XPO4/MALT1/SETD2/CTDSPL2/IPO11/ABCE1/UBR5/NUP155/NUP133	20
CC	GO:0031461	cullin-RING ubiquitin ligase complex	16/412	177/19518	1.15690088122635e-06	0.000480113865708937	0.000463978142891833	DCAF16/KLHDC1/FBXO25/DCAF1/AMN1/KCTD17/CUL2/SKP1/DCAF10/FBXW11/KLHDC2/ANAPC1/CUL4B/KLHL24/CRBN/FBXO3	16
CC	GO:0000151	ubiquitin ligase complex	18/412	307/19518	0.000100487533563452	0.0208511632144163	0.0201503948882501	DCAF16/KLHDC1/FBXO25/DCAF1/AMN1/KCTD17/CUL2/SKP1/DCAF10/FBXW11/KLHDC2/ARMC8/DCUN1D4/ANAPC1/CUL4B/KLHL24/CRBN/FBXO3	18
MF	GO:0140	ATP-dependent DNA damage	5/405	21/1183	7.72768286151	0.04631442563	0.04416644028	PMS2P1/PMS2P6/PMS2P5/PMS2P3/PMS2	5

	664	sensor activity		69	639e-05	49809	76577		
MF	GO:0140612	DNA damage sensor activity	5/405	24/18369	0.000152852889884426	0.0463144256349809	0.0441664402876577	PMS2P1/PMS2P6/PMS2P5/PMS2P3/PMS2	5
MF	GO:0019787	ubiquitin-like protein transferase activity	23/405	467/18369	0.000300906595566397	0.0469333643800541	0.0447566736922871	DZIP3/RNF103/G2E3/LNX2/FBXO25/KIAA1586/ZNF598/CUL2/MYCBP2/HERC4/FBXO22/LTN1/BIRC6/MALT1/FBXW11/HECTD1/BIRC3/RNF138/UBR5/UHRF2/TRIM3/FBXO3/RNF170	23
MF	GO:0004842	ubiquitin-protein transferase activity	22/405	438/18369	0.00030979118402676	0.0469333643800541	0.0447566736922871	DZIP3/RNF103/G2E3/LNX2/FBXO25/ZNF598/CUL2/MYCBP2/HERC4/FBXO22/LTN1/BIRC6/MALT1/FBXW11/HECTD1/BIRC3/RNF138/UBR5/UHRF2/TRIM3/FBXO3/RNF170	22

Supplementary Table 5 Gene Set Enrichment Analysis (GSEA) results showing pathways significantly associated with NDC1, NUP133, and TRMT11.

ID	Description	setSize	enrichmentSco	NES	pvalue	p.adjust	qvalue	rank	leading_edge	core_enrichme

					re							nt		
NDC1	KEGG_NEUROACTIVE_LIGAND_RECEPTOR_INTERACTION	KEGG_NEUROACTIVE_LIGAND_RECEPTOR_INTERACTION	KEGG_NEUROACTIVE_LIGAND_RECEPTOR_INTERACTION	272	-0.361080195	-2.068201147	1.37329350559563e-10	2.52686005029596e-08	1.64795220671476e-08	8489	tags=56%	list=40%	signal=34%	GABRB1/CHRNB4/FPR3/SSTR4/RXFP2/GABRA2/GHSR/GRM5/HTR1A/NPBWR1/P2RY6/GALR1/KISS1R/GALR3/GRM1/CHRNB1/GRIK3/GABRR2/GRID2/OPRK1/BDKRB1/LPAR4/P2RX7/GABRB2/DRD5/GRM3/GABRR1/AVPR1A/GABRA1/GRIK2/HTR1F/GABRB3/NPBWR2/ADORA3/GRM8/GRIK5/GCGR/MTNR1B/CHRNB3/HTR2C/GABBR2/GLRA3/P2RY2/GABRD/CRHR2/ADRA2B/CHRM2/ADORA1/LHCGR/MC2R/GRIN3A/HTR2A/TAAR2/CRHR1/OPRD1/ADRB1/PRSS3/P2RY4/CHRNA10/PTGER4/CSH1/CHRNE/HTR5A/AGTR1/LEPR/GABRG3/GRIN2C/CHRNB2/ADRA1D/PTGER3/C3AR1/GLP2R/CHRNA7/F2/THRB/BDKRB2/GLRA2/DRD2/GH2/GRIN2D/HTR1B/HCRTR1/HRH3/UTS2R/CHRM3/GABBR1/NPFFR2/LEP/MTNR1A/FSHB/TSHB/NMUR2/GRM4/LHB/GRIN1/OXTR/GPR50/GH1/GRIA3/CCKBR/PRL/PTGER1/PARD3/GALR2/GRID1/SSTR2/NMBR/GRIA2/TAAR9/PTH1R/VIPR1/CHRNG/ADRA2C/CHRNA4/CHRNA2/P2RY13/DRD4/GABRA5/GRIN3B/TACR2/F2RL1/S1PR3/NTSR1/CHRND/GPR35/P2RX4/SSTR1/APLNR/CHRM1/PTGFR/ADRA2A/P2RX2/HTR7/GLRA1/P2RY1/LTB4R2/LTB4R/F2RL3/ADORA2B/FPR1/C5AR1/OPRL1/PTGIR/PTAFR/EDNRB/TBXA2R/HRH2/P2RX1/LPAR2/TSPO/FPR2
NDC1	KEGG_LYSOSOME	KEGG_LYSOSOME	KEGG_LYSOSOME	120	-0.444906	-2.330225	3.107729	2.859110	1.864637	2274	tags=4	list=1	signal=	CTSE/GLB1/GGA3/CTSK/GNS/LAMP1/PSAPL1/CLN3/ARSB/GALNS/CLTCL1/PSAP/NAGLU/NAGPA/HYAL1/GNPTG/CLTB/DNASE2/ARSA/SORT1/GLA/LAPTM5/AP1S1/AP1B1/CTSZ/PPT1/CTSB/ATP6AP1/ATP6V0A1/ATP6V0D1/IDUA/SGSH/NAGA/GGA1/SLC11A1/NAPSA/MAN2B1

					5884	5679	08562665e-09	75877652e-07	45137599e-07		4%	1%	40%	/MCOLN1/ACP2/CTNS/CD63/CTSH/GM2A/GAA/ATP6V0B/ATP6V0C/NEU1/TCIRG1/CD68/TPP1/CTSD/PLA2G15/CTSA
NDC1	KEGG_SPLICEOSOME	KEGG_SPLICEOSOME	KEGG_SPLICEOSOME	126	0.452442377534194	2.14426940687312	9.88640761342988e-08	6.06366333623237e-06	3.95456304537195e-06	5548	tags=54%	list=26%	signal=40%	THOC3/THOC1/SF3B1/AQR/SRSF10/NCBP1/DDX46/DHX15/SRSF1/RBM25/SNRNP200/PRPF38B/SF3B3/U2SURP/LSM8/TRA2A/HSPA8/PRPF18/HNRNPA1L2/SRSF3/MAGOHB/SRSF8/SRSF7/SF3A3/NCBP2/TCERG1/SRSF5/THOC2/RBMX/HNRNPA3/PRPF3/PRPF4/PPIH/DDX42/RBM17/CDC5L/HNRNPA1/PRPF40A/DDX39B/PRPF38A/RBM8A/SNU13/PRPF8/SNRPA1/SLU7/SNRPF/SNRNP40/PRPF19/USP39/SNRPD1/PHF5A/SNRPD3/SRSF4/HNRNPU/HSPA1L/LSM6/SRSF9/BCAS2/SRSF2/HNRNPK/SMNDC1/DDX5/PPIL1/SRSF6/SNRPB2/PLRG1/PPIE/SNRPG
NDC1	KEGG_REGULATION_OF_ACTIN_CYTOSKELETON	KEGG_REGULATION_OF_ACTIN_CYTOSKELETON	KEGG_REGULATION_OF_ACTIN_CYTOSKELETON	211	-0.3441947	-1.909285	4.139069773	1.903972095	1.241720932	6009	tags=45%	list=28%	signal=3	FGF21/CHRM2/MYL10/FGD1/FGF3/ITGA2/ARPC5L/WASL/FGF19/INSRR/INS/GNG12/PIK3CD/PXN/IQGAP3/EGF/F2/HRAS/PDGFC/FGF13/FGF4/CRK/BDKRB2/MYH14/CHRM3/TIAM1/IQGAP1/FN1/TMSB4X/FGF17/GIT1/FGF12/FGF8/FGF2/BRAF/MYL7/FGF20/APC2/ITGB4/ARPC5/ACTG1/PIK3R5/WASF2/ARPC1B/ITGB3/ITGA5/MYLK/ARPC3/VCL/MSN/PDGFA/PAK1/ACTN4/M

					74	57	42089e-07	77361e-05	02627e-05				2%	YH9/ITGA7/CHRM1/MYL2/ARHGEF4/ITGB5/ITGA9/MAPK3/MAP2K2/FGF11/FGF1/PIP5K1C/ITGAX/PPP1CA/WASF1/DOCK1/RRAS/MYL5/SLC9A1/RAC2/ARAF/ACTB/LIMK1/PAK4/RAC1/SSH3/CSK/ITGA2B/WAS/ARPC4/CD14/MYL9/PFN1/CYFIP1/CFL1/ITGAM/ACTN1/GSN/ITGB2/LIMK2/RHOA
NDC1	KEGG_UBIQUITIN_MEDIATED_PROTEOLYSIS	KEGG_UBIQUITIN_MEDIATED_PROTEOLYSIS	KEGG_UBIQUITIN_MEDIATED_PROTEOLYSIS	129	0.432196832169381	2.0504299905412	5.47051719904149e-07	2.01315032924727e-05	1.31292412776996e-05	4441	tags=43%	list=21%	signal=35%	SMURF2/BIRC6/BIRC2/CUL4A/ANAPC1/HERC1/CBLB/CDC27/KLHL9/UBR5/XIAP/BTRC/HERC4/FBXW11/UBE2N/TRIM37/CUL4B/WWP1/ANAPC4/HERC2/CUL2/UBA6/CUL3/UBE3A/FBXW7/HERC3/CUL5/CUL1/BIRC3/UBE2K/ITCH/PIAS2/TRAF6/TRIP12/CDC23/ANAPC7/UBE2D2/FBXO4/DET1/NEDD4/SKP2/SKP1/UBE2G1/FANCL/PIAS1/UBE2Q2/UBE4A/DDB1/UBA2/CDC16/UBE3C/HUWE1/MAP3K1/TRIM32/ANAPC10/PRPF19
NDC1	KEGG_FC_GAMMA_R_MEDIATED_PHAGOCYTOSIS	KEGG_FC_GAMMA_R_MEDIATED_PHAGOCYTOSIS	KEGG_FC_GAMMA_R_MEDIATED_PHAGOCYTOSIS	96	-0.425198939	-2.138273743	3.0469062534441	9.3438458438953	6.0938125068882	3526	tags=41%	list=17%	signal=34%	DNM1/PLA2G4A/AMPH/ARPC5/WASF3/DNM2/PIK3R5/WASF2/PLPP3/ARPC1B/ARPC3/SYK/ASAP1/PLD1/AKT2/PAK1/GAB2/MYO10/SPHK2/MAPK3/ASAP2/PIP5K1C/WASF1/RAC2/NCF1/FCGR1A/LIMK1/PLD2/RAC1/FCGR2A/WAS/ARPC4/SPHK1/HCK/VASP/CFL1/GSN/PRKCD/LIMK2

							3e-06	3e-05	6e-05					
NDC1	KEGG_ECM_RECEPTOR_INTERACTION	KEGG_ECM_RECEPTOR_INTERACTION	KEGG_ECM_RECEPTOR_INTERACTION	84	-0.436533545	-2.143570068	3.79894287181374e-06	9.98579269162469e-05	6.51247349453784e-05	6090	tags=51%	list=29%	signal=37%	TNC/AGRN/COL2A1/ITGA2/THBS2/COL5A3/HSPG2/DAG1/THBS3/LAMC3/LAMB4/LAMA3/FN1/CHAD/LAMA5/COL4A2/LAMC1/ITGB4/LAMC2/LAMA4/ITGB3/ITGA5/COL6A3/VTN/COMP/THBS1/ITGA7/LAMB2/TNXB/ITGB5/ITGA9/CD36/SDC4/IBSP/GP1BA/GP5/SDC2/GP6/ITGA2B/SDC3/GP9/GP1BB/VWF
NDC1	KEGG_CELL_CYCLE	KEGG_CELL_CYCLE	KEGG_CELL_CYCLE	123	0.414532320621197	1.95980174644421	1.09687702978848e-	0.0002522817168513	0.0001645315544682	4401	tags=38%	list=21%	signal=30%	ORC3/ATM/ORC2/RBL1/PRKDC/SMAD4/ANAPC1/CDC27/RBL2/TFDP2/ATR/SMAD2/CDC7/CDKN1B/BUB3/MCM7/RAD21/ORC4/ANAPC4/MCM6/SMC3/CDK6/MAD2L1/CUL1/PTTG2/CDC14A/E2F5/CCNH/WEE1/ORC5/CDK7/CDC23/ANAPC7/CCND2/GSK3B/SKP2/MCM3/CDC25B/SKP1/HDAC2/STAG1/STAG2/HDAC1/CDC16/CDK2/CCNB3/ANAPC10

							05	49	71					
NDC1	KEGG_RNA_DEGRADATION	KEGG_RNA_DEGRADATION	KEGG_RNA_DEGRADATION	52	0.531615101356972	2.14733111344291	1.49338833405742e-05	0.000305314948296184	0.000199118444540989	4409	tags=54%	list=21%	signal=43%	XRN1/CNOT6L/CNOT1/CNOT9/PARN/DDX6/EXOSC10/PAPOLG/ZCCHC7/EXOSC2/LSM8/CNOT4/EXOSC3/DIS3/EXOSC9/HSPA9/CNOT2/PNPT1/EDC3/EXOSC7/CNOT6/EXOSC8/PAPOLA/CNOT10/CNOT7/ENO2/DCP2/HSPD1
NDC1	KEGG_COMPLEMENT_AND_COAGULATION_CASCADES	KEGG_COMPLEMENT_AND_COAGULATION_CASCADES	KEGG_COMPLEMENT_AND_COAGULATION_CASCADES	69	-0.453960716	-2.131283237	2.25777672603842e-05	0.000415430917591068	0.00027093320712461	5383	tags=48%	list=25%	signal=36%	C4BPB/C3AR1/F2/PLAT/BDKRB2/C9/MASP1/FGB/C4B/C4A/F7/FGA/KNG1/CFB/TFPI/SERPINF2/F8/F3/SERPINA1/SERPINE1/C2/PROS1/F13A1/F12/THBD/F5/PLAUR/C5AR1/C1QC/VWF/CR1/C1QB/C1QA

NDC1	KEGG_DILATED_CARDIOMYOPATHY	KEGG_DILATED_CARDIOMYOPATHY	KEGG_DILATED_CARDIOMYOPATHY	90	-0.409686869	-2.040022933	3.40177931681929e-05	0.000569024903904318	0.000371103198198468	3974	tags=39%	list=19%	signal=32%	CACNA2D3/ADCY4/SGCA/TNNI3/CACNG3/ITGB4/CACNG8/ACTG1/TPM1/CACNA1S/SLC8A1/CACNG1/ITGB3/ITGA5/ADCY3/DES/GNAS/TPM3/ACTC1/ITGA7/TNF/MYL2/TNNT2/IGF1/ITGB5/ITGA9/LMNA/MYBPC3/ACTB/TGFB1/CACNA2D4/ITGA2B/TPM4/RYR2/PRKACA
NDC1	KEGG_CHEMOKINE_SIGNALING_PATHWAY	KEGG_CHEMOKINE_SIGNALING_PATHWAY	KEGG_CHEMOKINE_SIGNALING_PATHWAY	187	-0.320544415	-1.751690305	4.48715132918625e-05	0.000688029870475225	0.000448715132918625	4571	tags=34%	list=22%	signal=27%	PPBP/PF4/GNG13/TIAM1/ADCY6/AKT1/PREX1/ADCY4/GRK2/RELA/GNG4/PARD3/CCL13/BRAF/CCL26/CXCR2/GNG8/STAT3/GRK6/GNG3/GNG5/PF4V1/PIK3R5/ADCY3/SHC2/PTK2B/AKT2/FOXO3/PAK1/NFKBIA/JAK3/CCR2/CCL19/CXCL1/CCL24/MAPK3/CCR10/IKBKG/PLCB3/GNB1/CCR1/GNAI2/CXCL2/GSK3A/RAC2/CXCL16/GNG10/NCF1/CXCL8/CCL3L3/CCL3/PLCB2/RAC1/CSK/WAS/FGR/ARRB2/GNB2/NFKBIB/PRKACA/HCK/GRB2/PRKCD/RHOA
ND	KEGG_AMINOAC	KEGG_AMINOAC	KEGG_AMINOAC	22	0.	2.	5.	0.	0.	45	ta	l	s	SEPSECS/FARS2/FARSB/MTFMT/IARS2/YARS2/NARS2/HARS2/WARS

C1	YL_TRNA_BIOSYNTHESIS	YL_TRNA_BIOSYNTHESIS	YL_TRNA_BIOSYNTHESIS		654254040757384	16932980346492	39469308373605e-05	000709016805291023	000462402264320233	03	gs=68%	ist=21%	ignal=54%	2/RARS2/MARS2/TARS2/LARS2/PSTK/DARS2
NDC1	KEGG_HYPERTROPHIC_CARDIOMYOPATHY_HCM	KEGG_HYPERTROPHIC_CARDIOMYOPATHY_HCM	KEGG_HYPERTROPHIC_CARDIOMYOPATHY_HCM	83	-0.408110611	-1.98767571	5.3727055358033e-05	0.000709016805291023	0.000462402264320233	4015	tags=40%	list=19%	signal=32%	PRKAG2/CACNA2D3/SGCA/PRKAA2/TNNI3/CACNG3/ITGB4/CACNG8/ACTG1/TPM1/CACNA1S/SLC8A1/CACNG1/ITGB3/ITGA5/DES/TPM3/ACTC1/ITGA7/TNF/MYL2/TNNT2/IGF1/ITGB5/ITGA9/LMNA/MYBPC3/ACTB/TGFB1/CACNA2D4/ITGA2B/TPM4/RYR2
NDC1	KEGG_CYTOKINE_CYTOKINE_RECEPTOR_INTE	KEGG_CYTOKINE_CYTOKINE_RECEPTOR_INTE	KEGG_CYTOKINE_CYTOKINE_RECEPTOR_INTE	262	-0.281	-1.596	6.4059	0.0007	0.0005	6317	tags=3	list=	signa	IFNL3/INHBA/BMP7/XCR1/INHBE/CCL22/TNFRSF13B/TNFSF9/CX3CL1/CCL17/IL15RA/IFNK/TNFSF14/CD70/IFNL2/LEPR/LIF/EGF/FLT3/IL10RA/PDGFC/VEGFC/GH2/TNFRSF8/IL18/PPBP/PF4/TNFR

	RACTION	RACTION	RACTION		288355	0861	3884879013e-05	8579516545159	12475107903211		5%	30%	1=25%	SF10D/LEP/IL22RA1/IL19/IL13/IL13RA1/GH1/IFNA8/PRL/ACVR1B/NGFR/CCL13/IL6R/TNFRSF10B/IL10RB/CCL26/IL1R2/CXCR2/EDA2R/PF4V1/TNFRSF14/VEGFA/IL25/IL17B/OSM/CSF1R/CTF1/IL12B/PDGFA/TNFRSF12A/TNF/CCR2/IFNGR1/TNFRSF4/IFNGR2/CCL19/CXCL1/IL1B/CCL24/CCR10/TNFSF13B/TNFRSF1B/IL10/CCR1/CXCL2/CXCL16/CXCL8/CCL3L3/TNFRSF6B/TGFB1/CCL3/CSF2RA/MPL/PLEKHO2/TNFRSF1A/IL17RA/CSF3R/ACVRL1/RELT/TNFRSF10C/CSF2RB/LTBR/EPOR/TNFSF12/TNFSF13
NDC1	KEGG_CALCIUM_SIGNALING_PATHWAY	KEGG_CALCIUM_SIGNALING_PATHWAY	KEGG_CALCIUM_SIGNALING_PATHWAY	177	-0.31532208	-1.710019968	7.1013433455083e-05	0.000816654484623346	0.000532600750841312	6054	tags=40%	list=29%	signal=29%	NOS2/CHRM2/LHCGR/HTR2A/CACNA1E/ADRB1/CACNA1B/HTR5A/AGTR1/GRIN2C/CALML5/ADRA1D/PTGER3/SLC8A3/CHRNA7/CAMK2B/BDKRB2/GRIN2D/CACNA1H/NOS1/CHRM3/GRIN1/OXTR/ADCY4/PHKG1/CCKBR/PTGER1/SLC8A2/PHKA1/CALML3/ITPKA/CACNA1S/SLC8A1/TACR2/MYLK/ADCY3/PLCE1/PTK2B/TNNC2/GNAS/NTSR1/P2RX4/CHRM1/PTGFR/P2RX2/RYR1/HTR7/SPHK2/GNA14/CAMK2A/PLCB3/LTB4R2/ADORA2B/SLC25A6/PLCD3/GNA11/PHKG2/PLCB2/PTAFR/EDNRB/SPHK1/CHP1/RYR2/TBXA2R/BST1/HRH2/GNAQ/P2RX1/PRKACA/CALM3/GNA15
NDC1	KEGG_LEISHMANIA_INFECTION	KEGG_LEISHMANIA_INFECTION	KEGG_LEISHMANIA_INFECTION	72	-0.4260	-2.0174	9.1270	0.0093	0.0060	2607	tags=3	list=1	signal	ELK1/PTPN6/HLA-DMA/IL12B/TNF/NFKBIA/MYD88/IFNGR1/IFNGR2/PTGS2/IL1B/JUN/MAPK3/IL10/FOS/IRAK1/NCF1/FCGR1A/TGFB1/FCGR2A/NCF4/N

					25729	43777	285371672e-05	1520736157709	7513523581115		8%	2%	=33%	CF2/NFKBIB/CR1/CYBA/ITGAM/ITGB2
NDC1	KEGG_LEUKOCYTE_TRANSENDOTHELIAL_MIGRATION	KEGG_LEUKOCYTE_TRANSENDOTHELIAL_MIGRATION	KEGG_LEUKOCYTE_TRANSENDOTHELIAL_MIGRATION	115	-0.357760293	-1.855524527	9.00459141955336e-05	0.000931520736157709	0.000607513523581115	2966	tags=32%	list=14%	signal=28%	ACTG1/CDH5/RAPGEF3/MMP9/PIK3R5/CLDN23/CLDN15/CLDN5/PTK2B/CLDN14/VCL/MSN/NOX3/NOX1/CYBB/ACTN4/MYL2/F11R/CTNNA1/ESAM/GNAI2/MYL5/RAC2/NCF1/ACTB/SIPA1/RAC1/NCF4/NCF2/MYL9/ICAM1/VASP/CYBA/ITGAM/ACTN1/ITGB2/RHOA
NDC1	KEGG_FOCAL_ADHESION	KEGG_FOCAL_ADHESION	KEGG_FOCAL_ADHESION	197	-0.2981823	-1.6465949	0.000100722	0.000975413	0.000636139	5213	tags=39%	list=25%	signal=3	CAV2/PIK3CD/PXN/EGF/HRAS/THBS3/PDGFC/CRK/VEGFC/LAMC3/LAMB4/LAMA3/AKT1/FLNC/FLNA/FN1/CHAD/CAPN2/LAMA5/COL4A2/BRAF/MYL7/TLN2/LAMC1/ITGB4/ACTG1/PIK3R5/VEGFA/LAMC2/ELK1/LAMA4/ITGB3/ITGA5/MYLK/COL6A3/SHC2/VCL/AKT2/VTN/COMP/PDGFA/THBS1/PAK1/ACTN4/ITGA7/LAMB2/MYL2/IGF1/TNXB/BAD/ITGB5/PARVG/JUN/ITGA9/MAP

					03	41	091010598	933997374	522172201				0%	K3/PIP5K1C/IBSP/SRC/PPP1CA/CCND3/DOCK1/MYL5/RAC2/ACTB/ILK/PAK4/RAC1/PARVB/ITGA2B/MYL9/VWF/GRB2/VASP/ACTN1/ZYX/TLN1/RHOA
NDC1	KEGG_PATHOGENIC_ESCHERICHIA_COLI_INFECTION	KEGG_PATHOGENIC_ESCHERICHIA_COLI_INFECTION	KEGG_PATHOGENIC_ESCHERICHIA_COLI_INFECTION	55	–0.447921814	–2.01787026	0.0001473508197600008	0.00135562754179207	0.000884104918560047	5968	tags=60%	list=28%	signal=43%	TUBA1B/TUBB4A/ARPC5L/WASL/TUBB/TUBA8/ARHGEF2/TUBAL3/TUBB1/HCLS1/TUBA1C/TLR4/TUBB6/KRT18/ARPC5/ACTG1/CTTN/TUBA3C/TUBA3E/ARPC1B/ARPC3/TUBB8/LY96/TUBB2A/TUBA3D/TLR5/ACTB/WAS/ARPC4/CD14/TUBA1A/TUBB4B/RHOA
NDC1	KEGG_CARDIAC_MUSCLE_CONTRACTION	KEGG_CARDIAC_MUSCLE_CONTRACTION	KEGG_CARDIAC_MUSCLE_CONTRACTION	73	–0.40099629	–1.906683423	0.000328091982	0.002874710702	0.001874811327	3306	tags=33%	list=16%	signal=28%	TNNI3/CACNG3/COX6B2/COX4I2/CACNG8/TPM1/CACNA1S/SLC8A1/CACNG1/COX6B1/COX8A/CYC1/UQCR11/ATP1A4/TPM3/ACTC1/FXYD2/MYL2/TNNT2/COX6A2/SLC9A1/CACNA2D4/TPM4/RYR2

							305013	10107	45722					
NDC1	KEGG_ENDOCYTOSIS	KEGG_ENDOCYTOSIS	KEGG_ENDOCYTOSIS	181	-0.302450837	-1.643955109	0.000401184173950027	0.00335535854576386	0.00218827731245469	5171	tags=41%	list=24%	signal=31%	VPS4A/NTRK1/EGF/HRAS/PARD6G/CHMP4C/CHMP3/SH3GL2/VPS37D/RAB11FIP1/SMAP2/PSD/ARFGAP1/NEDD4L/GRK2/SH3GLB2/GIT1/PSD4/VPS25/CBLC/PARD3/RUFY1/AP2A2/DNM1/RNF41/CXCR2/SH3GL3/GRK6/AP2A1/LDLR/IQSEC1/HLA-B/HLA-E/EHD1/DNM2/ARAP3/EPN3/CHMP2A/CSF1R/RAB11B/ASAP1/PSD2/PLD1/CLTCL1/HLA-A/DAB2/VPS28/HLA-G/HLA-C/CLTB/EHD2/EHD4/ACAP3/ASAP2/HSPA1B/HSPA1A/ARAP1/HGS/RAB31/PIP5K1C/SRC/CHMP4B/AP2M1/SH3GL1/PLD2/AP2S1/ARRB2/EHD3/IQSEC2/EPN1/HSPA6/RAB5C/VPS37C/MVB12A
NDC1	KEGG_DNA_REPLICATION	KEGG_DNA_REPLICATION	KEGG_DNA_REPLICATION	36	0.510307176841937	1.89874833326285	0.001108657060834	0.00871764804400	0.005685422637393	3495	tags=42%	list=17%	signal=35%	RFC5/RFC1/POLA1/RNASEH2B/MCM7/MCM6/POLA2/PRIM1/RPA1/RFC3/PRIM2/RNASEH1/MCM3/RPA3/DNA2

							06	41	98					
NDC1	KEGG_AXON_GUIDANCE	KEGG_AXON_GUIDANCE	KEGG_AXON_GUIDANCE	129	−0.320916712	−1.686193955	0.0011370845274788	0.0087176480440041	0.00568542263739398	2451	tags=23%	list=12%	signal=21%	EFNA4/UNC5A/EPHB6/SEMA3B/PAK1/PLXNB3/DCC/PLXNA2/UNC5B/MAPK3/EPHB2/CDK5/PLXNB2/EPHB3/ABLIM3/GNAI2/RAC2/DPYSL2/SEMA6B/LIMK1/PAK4/RAC1/EFNB1/SEMA4A/SEMA4B/CHP1/CFL1/FES/LIMK2/RHOA
NDC1	KEGG_VASCULAR_SMOOTH_MUSCLE_CONTRACTION	KEGG_VASCULAR_SMOOTH_MUSCLE_CONTRACTION	KEGG_VASCULAR_SMOOTH_MUSCLE_CONTRACTION	111	−0.32955464	−1.701416218	0.00124230202038549	0.00914334287003717	0.00596304969785033	4495	tags=32%	list=21%	signal=26%	MYH11/PLA2G2E/ADCY6/PLA2G2F/ADCY4/PRKG1/BRAF/PLA2G4A/CALML3/MYL6B/KCNMA1/RAMP3/CACNA1S/MYLK/ADCY3/PPP1R14A/GNAS/CALD1/KCNMB1/MYL6/MAPK3/MAP2K2/PPP1CA/PLCB3/ADORA2B/ARAF/GNA11/ARHGEF11/PLCB2/PTGIR/MYL9/GNAQ/PRKACA/PRKCD/CALM3/RHOA

NDC1	KEGG_INSULIN_SIGNALING_PATHWAY	KEGG_INSULIN_SIGNALING_PATHWAY	KEGG_INSULIN_SIGNALING_PATHWAY	136	-0.303935696	-1.605924082	0.00129482870807909	0.00916340316486744	0.00597613249882659	5222	tags=42%	list=25%	signal=32%	CALML5/PIK3CD/PRKAR2B/EIF4E1B/INSR/HRAS/CRK/RHOQ/INPP5K/G6PC2/AKT1/PRKAG2/PHKG1/CBLC/PPP1R3D/PDE3A/PRKAA2/BRAF/FASN/SREBF1/PHKA1/CALML3/PRKAR1A/PCK2/PIK3R5/IRS2/HK1/ELK1/PTPRF/SHC2/AKT2/PPP1R3B/GCK/IRS1/MKNK1/FBP1/TSC2/BAD/PYGL/MKNK2/EXOC7/MAPK3/MAP2K2/HK2/PPP1CA/GYS1/ARAF/PHKG2/EIF4EBP1/PRKACA/GRB2/CALM3/FLOT2/SH2B2/FLOT1/SOCS3/HK3
NDC1	KEGG_PPAR_SIGNALING_PATHWAY	KEGG_PPAR_SIGNALING_PATHWAY	KEGG_PPAR_SIGNALING_PATHWAY	69	-0.381156291	-1.789476457	0.00146450950532113	0.00966231036239473	0.00630150675808352	6182	tags=39%	list=29%	signal=28%	CPT1C/SLC27A5/CPT1A/ACOX1/FABP6/LPL/PPARD/AQP7/MMP1/ADIPOQ/NR1H3/PCK2/PPARG/APOA5/ANGPTL4/ACOX2/CD36/ACAA1/ME1/GK/ACSL1/ILK/RXRA/APOA2/SLC27A1/CYP27A1/PLTP
NDC1	KEGG_GAP_JUNCTION	KEGG_GAP_JUNCTION	KEGG_GAP_JUNCTION	87	-0.35	-1.73	0.001	0.009	0.006	5968	tags=	list	sign	TUBA1B/HTR2A/ADRB1/TUBB4A/TUBB/MAPK7/EGF/TUBA8/HRAS/PDGFC/TUBAL3/DRD2/ADCY6/TUBB1/ADCY4/GJD2/CSNK1D/TUBA1C/PRKG1/TUBB6/TUBA3C/TUBA3E/AD

					1173465	7022297	47035157688616	66231036239473	30150675808352		46%	=28%	al=33%	CY3/GNAS/PDGFA/TUBB8/TUBB2A/TUBA3D/MAPK3/MAP2K2/SRC/PLCB3/GNAI2/GNA11/PLCB2/GNAQ/PRKACA/TUBA1A/GRB2/TUBB4B
NDC1	KEGG_NUCLEOTIDE_EXCISION_REPAIR	KEGG_NUCLEOTIDE_EXCISION_REPAIR	KEGG_NUCLEOTIDE_EXCISION_REPAIR	44	0.476514163618038	1.85985368982701	0.00163217046579398	0.0103558401967618	0.00675380882397509	4981	tags=52%	list=24%	signal=40%	RFC5/RFC1/ERCC6/CUL4A/ERCC5/MNAT1/CUL4B/RPA1/RFC3/XPA/CCNH/CDK7/RPA3/GTF2H2/ERCC4/DDB1/GTF2H1/GTF2H5/RFC2/GTF2H3/XPC/ERCC8/ERCC3
NDC1	KEGG_RIBOSOME	KEGG_RIBOSOME	KEGG_RIBOSOME	87	0.36842802	1.64608770	0.00185434	0.01100645	0.00717812	6860	tags=52%	list=32%	signal=3	RPL3/RPL32/RPL37/RPL22/RPL9/RPS27L/RPL37A/RPL13/RSL24D1/RPL4/RPS3A/RPL7A/RPS17/RPSA/RPL5/RPL27A/RPL14/RPL35A/RPS18/RPS25/RPL15/RPS27A/RPL21/RPS10/RPS3/RPS6/RPS12/RPL31/RPL30/RPS29/RPS4Y1/RPL23/RPL6/RPL7/RPL10A/RPS7/RPS21/RPL22L1/RPL29/RPL36AL/

					8262313	304901	784283524	17123124	068194288				5%	RPL34/RPL23A/RPS15A/RPL13A/RPL39
NDC1	KEGG_CELL_ADHESION_MOLECULES_CAMS	KEGG_CELL_ADHESION_MOLECULES_CAMS	KEGG_CELL_ADHESION_MOLECULES_CAMS	133	-0.304581185	-1.610670577	0.00179945928759448	0.0110064517123124	0.00717812068194288	5528	tags=40%	list=26%	signal=30%	CLDN4/ALCAM/MADCAM1/HLA-DRB1/CD86/CLDN9/HLA-DRB4/HLA-DRB3/CLDN10/MAG/CLDN3/HLA-DMB/ICAM3/CNTN2/MPZL1/SELPLG/NRXN2/CDH2/CLDN11/HLA-B/HLA-E/CADM3/CDH5/CLDN23/CD276/NRXN1/NECTIN1/CNTN1/SELL/CDH15/CLDN15/PTPRF/CLDN5/CLDN14/HLA-DMA/HLA-A/MPZ/HLA-G/HLA-C/SELP/VCAN/F11R/ITGA9/SDC4/SDC2/CD4/PVR/ESAM/SDC3/ICAM1/NECTIN2/ITGAM/ITGB2
NDC1	KEGG_NOTCH_SIGNALING_PATHWAY	KEGG_NOTCH_SIGNALING_PATHWAY	KEGG_NOTCH_SIGNALING_PATHWAY	47	-0.426452052	-1.823810243	0.00219797170881	0.01263833732571	0.00824239390807	4628	tags=45%	list=22%	signal=35%	JAG2/DLL4/RBPJ/MAML1/JAG1/NOTCH3/NUMBL/NOTCH1/DVL1/MFNG/CTBP2/RBPJL/LFNG/DTX4/RFNG/DVL3/MAML3/NCOR2/NUMB/PTCRA/NCSTN

						964	29	365			
NDC1	KEGG_MATURITY_ONSET_DIABETES_OF_THE_YOUNG	KEGG_MATURITY_ONSET_DIABETES_OF_THE_YOUNG	KEGG_MATURITY_ONSET_DIABETES_OF_THE_YOUNG	25	-0.535569744	-1.93154911	0.0024966579283173	0.0137860687409495	0.00899091439627143	6237	tags=68%	list=29%	signal=48%	PKLR/NEUROD1/PAX4/HHEX/HNF1A/NR5A2/INS/HES1/NKX6-1/PDX1/MAFA/NKX2-2/ONECUT1/GCK/HNF4A/BHLHA15/MNX1
NDC1	KEGG_GLYCOLYSIS_GLUCONEOGENESIS	KEGG_GLYCOLYSIS_GLUCONEOGENESIS	KEGG_GLYCOLYSIS_GLUCONEOGENESIS	61	-0.390377963	-1.784591396	0.00257501840199622	0.0137860687409495	0.00899091439627143	3129	tags=33%	list=15%	signal=28%	PCK2/PGAM2/ALDH2/HK1/PGK1/LDHA/GCK/GPI/FBP1/ENO1/TPI1/HK2/GAPDH/ACSS2/ALDH3B1/PKM/PGAM4/PGAM1/HK3/ALDOA

NDC1	KEGG_T_CELL_RECEPTOR_SIGNALING_PATHWAY	KEGG_T_CELL_RECEPTOR_SIGNALING_PATHWAY	KEGG_T_CELL_RECEPTOR_SIGNALING_PATHWAY	108	0.349906654841954	1.61375414235991	0.00262235003224583	0.0137860687409495	0.00899091439627143	3238	tags=31%	list=15%	signal=27%	PIK3R1/NFATC3/CBLB/PIK3CA/AKT3/PPP3CC/RASGRP1/PTPRC/SOS1/PRKCQ/ICOS/MALT1/NFAT5/MAPK9/ITK/MAP3K7/CD28/PPP3CB/DLG1/FYN/CD40LG/PLCG1/CTLA4/CD3G/CHUK/CARD11/NFATC2/LCK/MAPK1/GSK3B/KRAS/CD247/IFNG/NFKB1
NDC1	KEGG_MISMATCH_REPAIR	KEGG_MISMATCH_REPAIR	KEGG_MISMATCH_REPAIR	23	0.564413924016128	1.89719734433889	0.00278167414424034	0.0139552832339472	0.00910127167431337	4052	tags=52%	list=19%	signal=42%	RFC5/RFC1/MSH2/PMS2/RPA1/RFC3/MLH3/MLH1/MSH6/RPA3/MSH3/SSBP1
NDC1	KEGG_HEMATOPOIETIC_CELL_	KEGG_HEMATOPOIETIC_CELL_	KEGG_HEMATOPOIETIC_CELL_	87	−0.33	−1.67	0.002	0.013	0.009	3492	tags=	list	sign	IL6R/IL1R2/CD1A/THPO/CD1D/ITGB3/ITGA5/CSF1R/ANPEP/TNF/IL1B/CD36/GP1BA/GP5/CD4/FCGR1A/CSF2RA/ITGA2B/GP9/CSF3R

	LINEAGE	LINEAGE	LINEAGE		9012068	6848625	80622543291329	9552832339472	10127167431337		30%	=17%	al=25%	/GP1BB/CD14/CD33/CR1/ITGAM/EPOR
NDC1	KEGG_ARRHYTHMOGENIC_RIGHT_VENTRICULAR_CARDIOMYOPATHY_ARVC	KEGG_ARRHYTHMOGENIC_RIGHT_VENTRICULAR_CARDIOMYOPATHY_ARVC	KEGG_ARRHYTHMOGENIC_RIGHT_VENTRICULAR_CARDIOMYOPATHY_ARVC	74	-0.352434342	-1.67697933	0.00297283907242068	0.0143947997190896	0.00938791286027582	3974	tags=51%	list=19%	signal=42%	CACNG6/CTNNA3/JUP/CACNA1C/SGCG/GJA1/DSG2/CACNG4/ITGA2/EMD/DAG1/CTNNA2/TCF7L1/CACNA2D3/SGCA/CDH2/CACNG3/ITGB4/CACNG8/ACTG1/CACNA1S/SLC8A1/CACNG1/ITGB3/ITGA5/DES/ACTN4/ITGA7/DSC2/CTNNA1/ITGB5/ITGA9/LMNA/ACTB/CACNA2D4/ITGA2B/RYR2/ACTN1
NDC1	KEGG_GLUTATHIONE_METABOLISM	KEGG_GLUTATHIONE_METABOLISM	KEGG_GLUTATHIONE_METABOLISM	50	-0.3936318	-1.7141413	0.00523019	0.02467579	0.01609291	4651	tags=36%	list=22%	signal=2	SMS/GGT5/GSR/GPX4/GSTZ1/MGST2/GPX2/IDH1/ANPEP/GSTA3/SRM/GPX1/GSTP1/MGST1/OPLAH/GGT1/G6PD/PGD

					59	02	621897717	75459436	14430067				8%	
NDC1	KEGG_PRION_DISEASES	KEGG_PRION_DISEASES	KEGG_PRION_DISEASES	35	-0.439184738	-1.71986854	0.0054101521547054 4	0.0247408288310692	0.0161353231506973	3776	tags=40%	list=18%	signal=33%	HSPA5/LAMC1/EGR1/NOTCH1/ELK1/IL1B/MAPK3/MAP2K2/HSPA1A/BAX/C1QC/PRKACA/C1QB/C1QA
NDC1	KEGG_VIBRIO_CHOLERAE_INFECTION	KEGG_VIBRIO_CHOLERAE_INFECTION	KEGG_VIBRIO_CHOLERAE_INFECTION	54	-0.38081059	-1.697686506	0.00551290207648	0.02474082883106	0.01613532315069	1376	tags=39%	list=7%	signal=36%	ATP6V1B2/SEC61A1/ATP6V1A/MUC2/ATP6V1G3/ACTG1/ADCY3/GNAS/KDELR1/KCNQ1/ATP6V1E1/ATP6AP1/ATP6V0A1/ATP6V0D1/ACTB/ATP6V1F/PRKACA/ATP6V0B/ATP6V0C/TCIRG1/ARF1

							824	92	73				
NDC1	KEGG_ARACHIDONIC_ACID_METABOLISM	KEGG_ARACHIDONIC_ACID_METABOLISM	KEGG_ARACHIDONIC_ACID_METABOLISM	57	-0.366449693	-1.642730311	0.00872274894564994	0.038213947761895	0.0249221398447141	4515	tags=33%	list=21%	signal=26%	GGT5/PLA2G2E/ALOX15B/PLA2G2F/LTC4S/GPX4/CYP4F2/PLA2G4A/PTGES2/CYP4F3/GPX2/TBXAS1/PTGS2/LTA4H/GPX1/ALOX12/PTGS1/ALOX5/GGT1
NDC1	KEGG_AMINO_SUGAR_AND_NUCLEOTIDE_SUGAR_METABOLISM	KEGG_AMINO_SUGAR_AND_NUCLEOTIDE_SUGAR_METABOLISM	KEGG_AMINO_SUGAR_AND_NUCLEOTIDE_SUGAR_METABOLISM	42	-0.398696064	-1.65506691	0.00902496956203946	0.0386184744050061	0.0251859615684822	2824	tags=36%	list=13%	signal=31%	HK1/NANS/GNPDA1/GCK/GPI/RENBP/HK2/GALE/NPL/CYB5R1/NAGK/CYB5R3/GMPPA/GALK1/HK3

NDC1	KEGG_BLADDER_CANCER	KEGG_BLADDER_CANCER	KEGG_BLADDER_CANCER	41	-0.409213151	-1.682297661	0.00954076617161827	0.0398977494449491	0.0260202713771407	5032	tags=41%	list=24%	signal=32%	EGF/HRAS/TP53/VEGFC/MMP1/BRAF/E2F2/MMP9/VEGFA/E2F3/THBS1/MAPK3/MAP2K2/CDKN1A/ARAF/CXCL8/DAPK1
NDC1	KEGG_EPITHELIAL_CELL_SIGNALING_IN_HELICOBACTER_PYLORI_INFECTION	KEGG_EPITHELIAL_CELL_SIGNALING_IN_HELICOBACTER_PYLORI_INFECTION	KEGG_EPITHELIAL_CELL_SIGNALING_IN_HELICOBACTER_PYLORI_INFECTION	68	-0.342530333	-1.604671612	0.011399315957025	0.0463926446716031	0.0302560726119151	2056	tags=26%	list=10%	signal=24%	PAK1/F11R/NFKBIA/CXCL1/JUN/SRC/IKBKG/ATP6V1E1/ATP6AP1/ATP6V0A1/ATP6V0D1/CXCL8/RAC1/CSK/ATP6V1F/ATP6V0B/ATP6V0C/TCIRG1
NDC1	KEGG_OOCYTE_MEIOSIS	KEGG_OOCYTE_MEIOSIS	KEGG_OOCYTE_MEIOSIS	112	0.3176	1.4691	0.0115	0.0463	0.0302	4184	tags=3	list=	signa	PPP2R5C/PPP2R1B/ANAPC1/CDC27/PPP3CC/BTRC/CAMK2D/PRKACB/FBXW11/SLK/ANAPC4/SMC3/MAD2L1/SGO1/FBXO5/CUL1/PTTG2/CALM1/PPP3CB/PPP2CA/CAMK2G/PPP2R5A/CDC23/ANAPC7/MAPK1/R

					56293369962	2092938568	981611679008	926446716031	560726119151		0%	20%	1=24%	PS6KA3/SKP1/ITPR2/ITPR3/ITPR1/IGF1R/PRKX/CDC16/CDK2
NUP133	KEGG_RIBOSOME	KEGG_RIBOSOME	KEGG_RIBOSOME	87	0.597774643678099	3.07667451182282	1e-10	6.13333333333333e-09	4.31578947368421e-09	5720	tags=67%	list=27%	signal=49%	RSL24D1/RPS27A/RPL30/RPL31/RPS27L/RPS29/RPL7A/RPL9/RPS3A/RPL37A/RPS10/RPL34/RPL15/RPL37/RPS7/RPSA/RPL6/RPS18/RPL21/RPL5/RPL14/RPL22/RPL17/RPS6/RPLP0/RPL7/RPS21/RPL35A/RPL36AL/RPL27A/RPS25/RPL13/RPS15A/RPL24/RPLP2/RPL3/RPL36A/RPL32/RPL22L1/RPS17/RPL23/RPS24/RPL10A/MRPL13/RPL4/RPL39/RPL12/RPS13/RPS4X/RPS27/RPS11/RPL13A/RPS26/RPL23A/RPL35/RPL27/RPS3/UBA52
NUP133	KEGG_SPLICEOSOME	KEGG_SPLICEOSOME	KEGG_SPLICEOSOME	126	0.526071241	2.870143779	1e-10	6.133333333	4.315789473	4947	tags=58%	list=23%	signal=4	AQR/U2SURP/SF3B1/NCBP1/THOC2/HNRNPA1L2/PRPF40A/THOC1/DHX15/DDX46/SRSF10/RBM25/LSM8/DDX42/HNRNPA1/HNRNPA3/PRPF18/SLU7/SRSF1/CDC5L/SRSF3/MAGOHB/PRPF38B/PRPF38A/HNRNPU/SRSF2/DDX5/SNRNP40/SNRPA1/RBMX/SRSF5/HNRNPK/LSM6/SRSF6/SF3A3/RBM17/SMNDC1/SNW1/SNRPD3/THOC3/PLRG1/CDC40/PRPF3/SF3B3/PRPF4/PPIH/SRSF7

					296725	07748		33333e-09	68421e-09				5%	/SNRPG/HSPA1L/SNRPD1/NCBP2/SNRPF/USP39/BCAS2/DDX39B/SNRNP200/RBM8A/SNRPE/ISY1/SNRPB2/HSPA8/TCERG1/HNRNPM/CWC15/SRSF8/SYF2/TXNL4A/PRPF8/DHX8/LSM3/LSM5/SNRNP27/PPIL1
NUP133	KEGG_NEUROACTIVE_LIGAND_RECEPTOR_INTERACTION	KEGG_NEUROACTIVE_LIGAND_RECEPTOR_INTERACTION	KEGG_NEUROACTIVE_LIGAND_RECEPTOR_INTERACTION	272	−0.37806567	−2.331874989	1e−10	6.13333333333333e−09	4.31578947368421e−09	9242	tags=62%	list=44%	signal=36%	NPY2R/CHRNA3/ADORA3/GABRQ/S1PR5/HTR1E/AGTR2/CTSG/TAAR6/GRIN2A/HCRTR2/GABRB2/P2RY8/ADCYAP1R1/P2RX3/CHRNB3/GABRR1/MC2R/GABRR2/FSHB/GABRG2/CHRNA2/GRIK2/GLRA2/ADRB3/HTR6/GRID2/GABRA2/THRB/PTGER3/GRM7/TAAR5/NTSR2/CHRM2/GHRHR/HTR2C/GLRA1/BRS3/EDNRB/CHRNA7/HTR1F/TSHB/TACR1/UTS2R/MC1R/GLP2R/AVPR1B/SCTR/EDNRA/AGTR1/P2RX6/AVPR2/GABRA5/NPY4R/GABRA1/P2RY1/SSTR5/F2RL1/CHRNB2/GH1/CHRNB4/GRIN3A/NPFFR1/MCHR1/GALR3/OPRM1/CHRNB1/TAAR2/LHCGR/MCHR2/TAAR9/ADORA2B/HTR1A/DRD3/CRHR2/SSTR4/GIPR/P2RX1/FPR2/GRM2/BDKRB2/PRLHR/GLRB/PRL/OPRD1/MTNR1B/CHRNA4/PRSS3/GRM1/VIPR1/NPFFR2/CSH1/HTR1B/GRIK5/OPRK1/GRM5/P2RY6/GABBR1/GPR50/PTGER4/GRM8/GABRD/CRHR1/GABBR2/P2RY2/SSTR3/GALR1/MTNR1A/TACR2/NPBWR1/ADRA1D/ADRA2A/GRIA2/NTSR1/F2RL3/FPR1/P2RY4/PTGIR/APLNR/GRID1/CHRND/ADRB1/HRH3/GRIK3/HRH2/GRM4/CCKBR/HTR7/PTH1R/GRIN1/KISS1R/GH2/GABRB3/P2RY11/TBXA2R/LHB/GCGR/GALR2/DRD5/SSTR2/CHRNA10/TSPO/PTAFR/NMBR/LEP/CHRM1/GABRG3/ADORA1/ADRA2B/GRIN2C/ADRA2C/F2/S1PR3/SSTR1/OPRL1/C5AR1/PTGER1/CHRNG/HCRTR1/GRIN2D/C

														HRNE/S1PR2/GPR35/LTB4R2/LTB4R/LPAR2/DRD4/P2RX2/DRD2/GRIN3B
NUP133	KEGG_UBIQUITIN_MEDIATED_PROTEOLYSIS	KEGG_UBIQUITIN_MEDIATED_PROTEOLYSIS	KEGG_UBIQUITIN_MEDIATED_PROTEOLYSIS	129	0.440373632610023	2.41367049707786	1.64995724699161e-09	7.58980333616141e-08	5.34065108894653e-08	3583	tags=40%	list=17%	signal=34%	BIRC6/CUL2/HERC4/ANAPC1/FBXW7/CUL3/UBR5/UBA6/CDC23/CUL5/HERC1/BIRC2/CUL4B/ANAPC4/BIRC3/UBE3A/WWP1/KLHL9/SMURF2/CDC16/SKP1/FBXW11/HERC3/RCHY1/ANAPC7/UBA3/XIAP/TRIP12/BTRC/CDC27/CUL4A/UBA2/ITCH/PIAS2/UBE2K/UBE2Q2/UBE2N/FBXO4/TRIM37/CUL1/NEDD4/ANAPC10/TRAF6/FANCL/UBE2E1/HERC2/UBE2G1/ERCC8/ANAPC5/SKP2/UBOX5/DET1
NUP133	KEGG_CELL_CYCLE	KEGG_CELL_CYCLE	KEGG_CELL_CYCLE	123	0.41071447415514	2.23192037908267	1.10550328134175e-	4.06825207533763e-	2.86267165484284e-	3455	tags=36%	list=16%	signal=30%	ATR/SMAD2/ANAPC1/ORC3/PRKDC/ORC2/SMC3/ORC4/CDC23/RAD21/ATM/ORC5/STAG2/ANAPC4/BUB3/RBL1/CDC16/CCNH/SKP1/MAD2L1/ANAPC7/STAG1/CDC14A/E2F5/HDAC2/CDC27/RBL2/HDAC1/CDK6/TFDP2/WEE1/SMAD4/CUL1/CDK7/PTTG2/ANAPC10/MCM6/GSK3B/YWHAB/ANAPC5/CDKN1B/SKP2/YWHAZ/MCM3

							07	06	06					
NUP133	KEGG_RNA_DEGRADATION	KEGG_RNA_DEGRADATION	KEGG_RNA_DEGRADATION	52	0.539949110289902	2.47803379134485	2.63727899458098e-07	8.0876558338166e-06	5.69097046199053e-06	4929	tags=63%	list=23%	signal=49%	ZCCHC7/DIS3/PARN/DDX6/LSM8/EXOSC9/XRN1/CNOT1/CNOT10/CNOT9/PAPOLG/CNOT2/CNOT6L/EXOSC7/EXOSC3/PNPT1/EXOSC8/LSM6/EXOSC10/CNOT6/EXOSC2/EDC3/PAPOLA/HSPA9/CNOT7/HSPD1/LSM1/EXOSC1/DCP2/CNOT4/ENO2/LSM3/LSM5
NUP133	KEGG_VALINE_LEUCINE_AND_ISOLEUCINE_DEGRADATION	KEGG_VALINE_LEUCINE_AND_ISOLEUCINE_DEGRADATION	KEGG_VALINE_LEUCINE_AND_ISOLEUCINE_DEGRADATION	43	0.516439619465055	2.28265664305184	1.20434214238986e-05	0.00031656993457105	0.000222758020321734	4062	tags=47%	list=19%	signal=38%	DBT/HIBADH/HIBCH/OXCT1/BCKDHB/MCCC1/MCCC2/ALDH9A1/HMGCS1/ACADSB/DLD/MCEE/ACADM/PCCA/ACAT1/HADH/AUH/PCCB/ALDH3A2/ACAT2

NUP133	KEGG_PROTEASOME	KEGG_PROTEASOME	KEGG_PROTEASOME	43	0.511493588506476	2.26079524822696	1.58108347340491e-05	0.000363649198883129	0.000255885877932636	4428	tags=47%	list=21%	signal=37%	PSME4/PSMD12/PSMA5/PSMD14/PSMA3/PSMD13/PSMB7/PSMC6/PSMA4/PSMA2/PSMD6/PSMD1/PSMA6/PSMB9/IFNG/PSMA1/PSMC1/PSMB1/PSMB5/PSMC2
NUP133	KEGG_MATURITY_ONSET_DIABETES_OF_THE_YOUNG	KEGG_MATURITY_ONSET_DIABETES_OF_THE_YOUNG	KEGG_MATURITY_ONSET_DIABETES_OF_THE_YOUNG	25	-0.597743018	-2.278847629	3.29318284827985e-05	0.000673272937870547	0.000473756129050785	6282	tags=76%	list=30%	signal=53%	FOXA2/PAX4/HNF1A/NEUROG3/FOXA3/HNF1B/NR5A2/PKLR/PDX1/GCK/HES1/HNF4A/NKX2-2/BHLHA15/INS/NKX6-1/MAFA/MNX1/ONECUT1
NU	KEGG_PROTEIN	KEGG_PROTEIN	KEGG_PROTEIN	23	0.	2.	3.	0.	0.	55	ta	l	s	SRP54/SEC62/IMMP2L/SRP9/SEC63/SPCS3/SRP72/SEC61A2/SRPR

P133	_EXPORT	_EXPORT	_EXPORT		641432956724409	34741259308374	71685243094636e-05	000683900847294131	000481234577901476	70	gs=74%	ist=26%	ignal=55%	B/SRP68/IMMP1L/SPCS2/SEC61G/SPCS1/SEC11C/SRPRA/SEC11A
NUP133	KEGG_ECM_RECEPTOR_INTERACTION	KEGG_ECM_RECEPTOR_INTERACTION	KEGG_ECM_RECEPTOR_INTERACTION	84	-0.380618614	-1.952082793	4.69726423897228e-05	0.000785724199973545	0.00055288373339095	7121	tags=55%	list=34%	signal=36%	COL6A6/ITGA3/THBS2/THBS3/COL4A6/COL5A2/GP1BB/GP5/LAMA1/COL11A2/COL3A1/GP9/COL6A3/DAG1/COL11A1/COL1A1/ITGA11/TNC/LAMC3/ITGA7/VWF/LAMA4/SDC1/RELN/COL1A2/AGRN/COL5A1/ITGA9/COL4A1/COL2A1/LAMA5/VTN/COL6A1/CHAD/IBSP/SDC3/ITGA5/COL4A2/LAMA3/LAMB2/HSPG2/COMP/COL5A3/SDC2/ITGB4/TNXB
NUP1	KEGG_OOCYTE_MEIOSIS	KEGG_OOCYTE_MEIOSIS	KEGG_OOCYTE_MEIOSIS	112	0.3651	1.9468	6.0195	0.0009	0.0006	3419	tags=3	list=	signa	ANAPC1/PPP3CB/SMC3/CDC23/ITPR2/RPS6KA3/ANAPC4/PPP2R1B/PPP2CA/CDC16/SKP1/FBXW11/MAD2L1/PRKACB/ANAPC7/PPP3CC/PPP1CC/CALM1/BTRC/CDC27/PPP2R5A/SLK/PPP3R1/CAMK2D/PPP3C

33					63790588927	977659036	6100849312e-05	22999354635611	49478950916363		4%	16%	1=29%	A/PPP2R5C/CUL1/PTTG2/ANAPC10/PPP1CB/FBXO5/ITPR1/SGO1/YWHAB/ANAPC5/CALM2/MAPK1/YWHAZ
NUP133	KEGG_NUCLEOTIDE_EXCISION_REPAIR	KEGG_NUCLEOTIDE_EXCISION_REPAIR	KEGG_NUCLEOTIDE_EXCISION_REPAIR	44	0.491387346571093	2.18144502021608	9.47858416331803e-05	0.00134158422003886	0.000944020932864873	3934	tags=48%	list=19%	signal=39%	RFC1/ERCC6/CUL4B/XPA/ERCC5/GTF2H1/CCNH/CUL4A/ERCC4/MNAT1/CDK7/RFC4/RPA1/GTF2H3/RFC3/RFC5/ERCC8/GTF2H2/RFC2/GTF2H5/XPC
NUP133	KEGG_PROPANOATE_METABOLISM	KEGG_PROPANOATE_METABOLISM	KEGG_PROPANOATE_METABOLISM	31	0.5381644	2.1602489	0.0001178	0.0015484	0.0010896	4344	tags=45%	list=2	signal=3	SUCLA2/HIBCH/ALDH9A1/MCEE/ACADM/PCCA/ACAT1/ACACA/LDHB/PCCB/SUCLG2/ALDH3A2/ACAT2/SUCLG1

					27990736	1238736	1976703951	8836680499	1137938795			1%	6%	
NUP133	KEGG_AMINOACYL_TRNA_BIOSYNTHESIS	KEGG_AMINOACYL_TRNA_BIOSYNTHESIS	KEGG_AMINOACYL_TRNA_BIOSYNTHESIS	22	0.617243112020548	2.23919793612895	0.000172885172998631	0.0021207247887832	0.00149227201956713	3006	tags=55%	list=14%	signal=47%	FARSB/IARS2/SEPSECS/YARS2/FARS2/NARS2/RARS2/WARS2/MTFMT/HARS2/DARS2/LARS2
NUP133	KEGG_MISMATCH_REPAIR	KEGG_MISMATCH_REPAIR	KEGG_MISMATCH_REPAIR	23	0.605740881754	2.21679250975	0.000207225273	0.002333492431	0.001641988381	3531	tags=57%	list=17%	signal=47%	MSH2/RFC1/MLH1/SSBP1/MSH6/PMS2/MSH3/MLH3/RFC4/RPA1/RFC3/RFC5/RFC2

					143	29	8423887	39262	35751		
NUP133	KEGG_REGULATION_OF_ACTIN_CYTOSKELETON	KEGG_REGULATION_OF_ACTIN_CYTOSKELETON	KEGG_REGULATION_OF_ACTIN_CYTOSKELETON	211	−0.278530111	−1.662729933	0.000215594409422145	0.00233349243139262	0.00164198838135751	5168	tags=36%, list=24%, signal=27%	ITGA11/CD14/FGF22/GSN/ACTN1/PAK6/FGF12/WASF2/WASF1/FGF8/ITGA7/BAIAP2/ITGAD/INSRR/MSN/FGF13/FGF16/FGF3/ACTN3/ITGAX/FGF18/ITGB2/FGF4/FGFR3/IQGAP3/MYH14/FGF17/FGFR4/BCAR1/MYL2/WASL/ITGA9/ACTN4/FGF2/FGF21/FGF1/PIK3R2/RAC2/CSK/PPP1CA/ACTB/PXN/ACTG1/PIK3R5/MAPK3/PIK3CD/RHOA/PFN3/ITGAM/MYLK3/INS/CHRM1/MYL7/RAC3/HRAS/APC2/ITGA5/FGF20/F2/LIMK2/ARAF/SLC9A1/CFL1/GIT1/ARHGEF4/FGF19/MAP2K2/ITGB4/LIMK1/SSH3/PIP5K1C/FGF11/WAS/PFN1/MYL5/PAK4
NUP133	KEGG_OLFACTORY_TRANSDUCTION	KEGG_OLFACTORY_TRANSDUCTION	KEGG_OLFACTORY_TRANSDUCTION	387	−0.233964277	−1.508149046	0.000247631806683	0.002531347357213	0.001781211241060	12026	tags=62%, list=57%, signal=27%	OR13J1/OR9G4/OR14I1/OR6C74/OR2T1/OR14C36/OR6N1/OR13D1/OR2G3/OR8B4/OR52I2/OR52M1/OR2M5/OR4Q3/OR52W1/OR51T1/OR5J2/OR2T2/OR5I1/OR13A1/OR4D11/OR13C2/OR4C12/OR1K1/OR1J1/OR10P1/OR5V1/OR52D1/OR5A1/OR9A4/OR5M11/OR5AR1/OR1N1/OR5M10/OR10S1/OR1D4/OR10J5/OR14A16/OR11A1/PDE1C/OR51I2/OR51V1/OR6B2/OR6V1/OR12D3/OR51Q1/GNAL/OR12D2/OR4C15/OR10X1/OR2A2/OR1N2/OR4D2/OR10H1/OR51B5/OR10G2/OR6C75/OR10A3/OR1J4/OR2A14/OR4C11/OR4M2/OR4F6/OR56A1/OR7C1/OR10J1/OR8J3/OR52R1/OR10H5/OR8S

	957	79	04	1/OR10A4/OR9A2/OR1L8/OR5AP2/OR2L13/OR6B3/OR10K1/OR5M1/OR5B12/OR1E1/OR2B6/OR52J3/OR52E6/OR51A4/OR3A1/OR9I1/OR13H1/OR2M3/OR5AK2/OR5M9/OR4X2/PRKG1/OR5L2/OR10R2/OR6C3/OR10Z1/OR4A5/OR5D14/OR9K2/CLCA4/OR4N2/OR1A1/OR10H4/OR2T4/OR10AD1/ADCY3/OR4K13/OR1D5/OR1M1/OR4N5/OR2F1/OR2AG1/OR5K2/OR4K15/OR52I1/OR5AN1/OR51F2/OR4S2/OR4K14/OR5K1/OR1G1/OR5H2/OR4D5/OR5C1/OR2C1/OR2B3/OR5AC2/OR10T2/OR13C5/OR51L1/OR51G1/OR51G2/OR4C45/OR5B17/OR2T33/OR2T12/OR5F1/OR5AU1/OR8D1/OR2W1/OR7A5/OR10V1/OR52K1/OR56A4/OR8D4/OR6T1/OR52N5/OR52L1/OR1L4/OR1C1/OR52B2/OR2T8/OR2A12/OR6A2/OR8J1/OR56B4/OR13C8/OR51F1/OR2M7/OR4C46/OR7G3/OR5A2/OR8D2/OR2H1/OR5M8/OR8A1/OR1E2/OR2D2/OR2S2/OR9Q1/GUCA1C/OR52N2/OR7A10/OR6K2/OR56A5/OR10H2/GUCA1B/OR10G3/OR8K5/PRKACG/OR13C9/OR4N4/OR6C68/OR5M3/OR10A2/OR10A5/OR1L6/OR10G8/OR6Y1/OR2B2/OR1A2/OR4B1/OR51A2/OR7D4/CNGA4/OR4C13/OR52K2/OR2H2/OR10Q1/OR56A3/OR8B8/OR4F17/OR4F5/OR4F4/OR9Q2/OR6K6/CLCA2/OR2C3/OR52N1/OR11L1/OR2J3/OR10G9/OR52E8/OR2W3/OR51E1/OR10G7/OR51E2/OR1I1/OR10C1/OR2G2/OR5D16/CNGA3/OR4D9/OR10G4/OR2J2/CAMK2A/CALM3/CAMK2B/PRKACA/OR7D2/OR2V2/OR2B11/OR1L3/CALML5/CALML3/OR2Y1/GUCA1A/CALML6/GUCY2D/CNGB1/ARRB2

NUP133	KEGG_CALCIUM_SIGNALING_PATHWAY	KEGG_CALCIUM_SIGNALING_PATHWAY	KEGG_CALCIUM_SIGNALING_PATHWAY	177	-0.283998342	-1.63702874	0.00039205511972552	0.00379674431734188	0.002671622145498	7927	tags=50%	list=37%	signal=32%	CACNA1D/PTGER3/ERBB2/NOS3/BST1/ATP2A3/CHRM2/EGFR/RYR3/PRKACG/HTR2C/ERBB4/EDNRB/CHRNA7/TACR1/CACNA1G/ATP2B3/PLCB4/ERBB3/AVPR1B/EDNRA/AGTR1/P2RX6/ADCY2/GNA14/PHKA1/LHCGR/CACNA1F/ADORA2B/P2RX1/CACNA1C/CACNA1A/BDKRB2/CHP2/ADCY7/ATP2B2/GRM1/CACNA1E/ATP2A1/GRM5/SLC25A6/CAMK2A/RYR2/CALM3/NOS1/TACR2/ADRA1D/NTSR1/CAMK2B/PHKG2/PRKACA/NOS2/CACNA1B/PLCE1/ADRB1/HRH2/RYR1/CCKBR/CALML5/HTR7/CALML3/GRIN1/TBXA2R/DRD5/PTK2B/CACNA1S/PTAFR/MYLK3/CALML6/CHRM1/SLC8A2/PHKG1/ITPKA/TNNC2/GRIN2C/GNA15/CACNA1H/PTGER1/PLCB2/SPHK1/GRIN2D/ADCY4/GNA11/LTB4R2/PLCB3/P2RX2/CHP1/PLCD3/SPHK2
NUP133	KEGG_DILATED_CARDIOMYOPATHY	KEGG_DILATED_CARDIOMYOPATHY	KEGG_DILATED_CARDIOMYOPATHY	90	-0.340593128	-1.747753933	0.000584685089301797	0.00537910282157654	0.00378506663074322	8479	tags=60%	list=40%	signal=36%	CACNG2/ADCY1/CACNB4/MYH7/ITGA10/ITGA8/CACNA1D/CACNG6/PRKACG/ITGA3/ADCY5/TPM2/ADCY2/CACNB2/CACNB3/CACNG4/ACTC1/EMD/CACNA1F/CACNA1C/CACNG3/DAG1/LMNA/SGCG/ITGA11/ADCY7/MYL3/CACNG8/ADCY6/IGF1/TNF/ITGA7/RYR2/TGFB1/CACNG7/MYH6/PRKACA/MYL2/ADRB1/ITGA9/SGCA/ACTB/ACTG1/CACNB1/CACNA1S/ITGA5/CACNG1/ADCY4/CACNA2D4/TNNT2/TNNI3/MYBPC3/ITGB4/DES

NUP133	KEGG_CYTOKINE_CYTOKINE_RECEPTOR_INTERACTION	KEGG_CYTOKINE_CYTOKINE_RECEPTOR_INTERACTION	KEGG_CYTOKINE_CYTOKINE_RECEPTOR_INTERACTION	262	-0.253214551	-1.547069446	0.000695409209034485	0.00609310926011168	0.00428748534893442	9038	tags=47%	list=43%	signal=27%	MET/CCL23/IL10RB/IFNA6/IL11RA/EDA2R/IL18/IL4/IFNA7/IL19/CCL8/CCL15/CCL14/BMPR1B/IL9/MPL/CCL25/TNFSF15/INHBA/IFNA8/IFNA14/IFNK/TNFRSF18/CSF1R/TNFSF13B/CXCL6/CXCR3/IL9R/IL2RG/EGFR/IL1R2/LIF/CXCL9/IL20RA/CCL16/TNFRSF13B/TNFSF14/INHBB/IFNGR2/CXCL5/CXCL3/CCL27/CXCL12/GH1/CCL17/IFNL1/BMP2/CCL3L3/KDR/LIFR/PDGFB/TNFRSF10D/FLT4/CX3CL1/CSF2/CCR1/PRL/AMHR2/TNFRSF10C/CXCR2/IL13/CCL3/CLCF1/CSF3/CCL19/CXCL14/IL10/IL10RA/IL1B/CXCL8/CXCL16/TNF/CCL13/CNTFR/CD70/CSF2RA/TNFRSF11A/EPO/TPO/IL4R/TGFB1/CCL24/TNFSF9/CCL22/VEGFA/XCR1/IL22RA1/CCL21/BMP7/CCL26/ACVRL1/IL12B/IL25/OSM/GH2/EPOR/IFNL3/LEP/CXCL2/IL3/NGFR/IL17B/IL11/CCR10/TNFRSF14/IL15RA/IL6R/PLEKHO2/LTBR/TNFRSF8/CSF2RB/TNFRSF4/AMH/CSF3R/TNFRSF12A/RELT/TNFRSF1B/CXCL1/TNFRSF1A/IL17RA/TNFRSF6B/TNFSF12/TNFSF13/CTF1
NUP133	KEGG_BASAL_TRANSCRIPTION_FACTORS	KEGG_BASAL_TRANSCRIPTION_FACTORS	KEGG_BASAL_TRANSCRIPTION_FACTORS	35	0.4725703764138	1.98243224531353	0.000955784815883	0.007993836641936	0.005624953701133	5337	tags=60%	list=25%	signal=45%	TAF2/GTF2E2/GTF2H1/TAF4B/TAF11/GTF2I/GTF2A1/GTF2B/TAF7/TBPL1/GTF2H3/TAF9/TAF5/GTF2H2/GTF2F2/TAF1L/GTF2E1/TAF1/GTF2A2/TAF9B/TAF13

					88		735	69	94			
NUP133	KEGG_DNA_REPLICATION	KEGG_DNA_REPLICATION	KEGG_DNA_REPLICATION	36	0.459768786838347	1.92721998305209	0.00142718874405857	0.0114175099524685	0.00803406020682855	3531	tags=42%, list=17%, signal=35%	RNASEH2B/RFC1/POLA1/SSBP1/PRIM2/RFC4/DNA2/RPA1/PRIM1/RFC3/RFC5/RNASEH1/MCM6/MCM3/RFC2
NUP133	KEGG_GLYCOSAMINOGLYCAN_DEGRADATION	KEGG_GLYCOSAMINOGLYCAN_DEGRADATION	KEGG_GLYCOSAMINOGLYCAN_DEGRADATION	21	−0.54333345	−1.965384168	0.00177379170304568	0.0135990697233502	0.00956913945064116	2441	tags=38%, list=12%, signal=34%	HS3ST3A1/HYAL3/IDUA/SGSH/HYAL2/GALNS/NAGLU/HYAL1

NUP133	KEGG_COMPLEMENT_AND_COAGULATION_CASCADES	KEGG_COMPLEMENT_AND_COAGULATION_CASCADES	KEGG_COMPLEMENT_AND_COAGULATION_CASCADES	69	-0.351743043	-1.729320536	0.00208907389250109	0.015375583848808	0.0108192037380057	8606	tags=57%	list=41%	signal=34%	SERPIND1/F9/SERPINC1/F11/MASP2/F13B/C1QB/CFI/C8B/PROC/KNG1/SERPINA5/C1QA/F10/MASP1/FGA/SERPINE1/C9/BDKRB2/C1QC/C2/C4B/C4A/PLAU/C4BPB/CFB/F7/VWF/THBD/F3/PLAT/C8G/FGB/C1R/PLAUR/SERPINF2/F2/C5AR1/F12
NUP133	KEGG_FOCAL_ADHESION	KEGG_FOCAL_ADHESION	KEGG_FOCAL_ADHESION	197	-0.254050737	-1.491580325	0.00219459957447097	0.0155310123731792	0.0109285727797542	6119	tags=41%	list=29%	signal=29%	LAMA1/DOCK1/COL11A2/PAK5/KDR/PDGFB/PARVG/COL3A1/FLT4/COL6A3/MYL10/JUN/COL11A1/COL1A1/CCND1/ITGA11/ACTN1/SHC3/TNC/PAK6/LAMC3/IGF1/ITGA7/VWF/LAMA4/RELN/ACTN3/VEGFA/COL1A2/BCAR1/TLN2/CAV3/MYL2/AKT1/COL5A1/ITGA9/COL4A1/ACTN4/COL2A1/LAMA5/PIK3R2/RAC2/VTN/PPP1CA/ACTB/PXN/ACTG1/PIK3R5/MAPK3/PIK3CD/RHOA/COL6A1/SRC/FLNA/CHAD/IBSP/MYLK3/MYL7/RAC3/HRAS/FLNC/ITGA5/TLN1/SHC2/CCND3/COL4A2/LAMA3/LAMB2/AKT2/ELK1/SHC1/COMP/COL5A3/ITGB4/BAD/PIP5K1C/ZYX/TNXB/VASP/MYL5/PAK4
NUP1	KEGG_NOTCH_SIGNALI	KEGG_NOTCH_SIGNALI	KEGG_NOTCH_SIGNALI	47	-0.39	-1.76	0.002	0.017	0.012	3784	tags=	list	sign	MAML3/CTBP1/HES5/DVL3/DVL2/LFNG/HES1/NCSTN/CTBP2/NOTCH4/PTCRA/DTX4/RBPJL/NOTCH1/N

33	NG_PATHWAY	NG_PATHWAY	NG_PATHWAY		979863	0862347	54836357034106	2804613089074	1595923397918		43%	=18%	al=35%	COR2/NOTCH3/RFNG/NUMBL/MAML1/DVL1
NUP133	KEGG_INSULIN_SIGNALING_PATHWAY	KEGG_INSULIN_SIGNALING_PATHWAY	KEGG_INSULIN_SIGNALING_PATHWAY	136	-0.286556225	-1.582754237	0.00262963541657286	0.0172804613089074	0.0121595923397918	3912	tags=34%	list=18%	signal=28%	CALM3/PCK2/HK2/SREBF1/PHKG2/PRKACA/HK3/PRKAG3/PKLR/AKT1/EIF4E1B/GCK/IRS2/PIK3R2/SLC2A4/CALML5/CALML3/PPP1CA/PIK3R5/MAPK3/CBLC/PIK3CD/PPP1R3D/CALML6/INS/PHKG1/HRAS/SOCS3/SHC2/PTPRF/RPS6KB2/FASN/ARAF/AKT2/MKNK2/TSC2/GYS1/ELK1/SHC1/FLOT1/MAP2K2/BAD/EIF4EBP1/EXOC7/FLOT2/SH2B2
NUP133	KEGG_BASAL_CELL_CARCINOMA	KEGG_BASAL_CELL_CARCINOMA	KEGG_BASAL_CELL_CARCINOMA	55	-0.3831159	-1.7700855	0.00295183	0.01872886	0.01317878	7351	tags=64%	list=35%	signal=4	GLI1/WNT3A/WNT5A/WNT8A/HHIP/TP53/WNT8B/FZD7/BMP2/WNT1/FZD5/SUFU/WNT9A/FZD9/TCF7L1/WNT11/WNT3/DVL3/SHH/GLI3/DVL2/WNT4/FZD10/FZD8/WNT9B/GLI2/APC2/WNT5B/WNT10A/SMO/FZD1/WNT6/BMP4/FZD2/DVL1

					79	55	262157199	90472154	08513014				2%	
NUP133	KEGG_MELANOGENESIS	KEGG_MELANOGENESIS	KEGG_MELANOGENESIS	101	-0.308145695	-1.628661503	0.00347635297573231	0.0213216315844915	0.0150032075794763	7302	tags=51%	list=35%	signal=34%	EDNRB/WNT3A/WNT5A/PLCB4/WNT8A/MC1R/WNT8B/FZD7/ADCY5/ADCY2/WNT1/TYR/CREBBP/CREB3L4/MITF/ADCY7/CREB3L1/FZD5/WNT9A/ADCY6/FZD9/CAMK2A/TCF7L1/CALM3/WNT11/WNT3/CAMK2B/DVL3/PRKACA/DVL2/WNT4/FZD10/CREB3L3/FZD8/CALML5/CALML3/WNT9B/MAPK3/CALML6/HRAS/WNT5B/WNT10A/GNAI2/FZD1/CREB3L2/WNT6/PLCB2/ADCY4/MAP2K2/PLCB3/FZD2/DVL1
NUP133	KEGG_AXON_GUIDANCE	KEGG_AXON_GUIDANCE	KEGG_AXON_GUIDANCE	129	-0.285582718	-1.567008779	0.00385176963936	0.02286211656916	0.01608718728836	5110	tags=39%	list=24%	signal=30%	CHP2/EFNB2/RGS3/SEMA3F/SEMA5B/PAK6/SEMA6C/DPYSL2/PLXNB2/PLXNB3/SEMA6D/EPHA6/EPHB2/EPHA2/EPHA8/PLXNA2/RAC2/DPYSL5/EFNA2/DCC/MAPK3/UNC5B/RHOA/EFNB3/SEMA3G/RAC3/HRAS/NGEF/SEMA4A/GNAI2/SEMA6B/PLXNB1/LIMK2/ROBO3/NFATC4/NTN3/UNC5A/SLIT3/EFNB1/SEMA3B/EPHB6/CFL1/NTN1/SEMA4G/EPHB3/EFNA3/LIMK1/CHP1/FES/PAK4

							…967	…19	…91					
NUP133	KEGG_HYPERTROPHIC_CARDIOMYOPATHY_HCM	KEGG_HYPERTROPHIC_CARDIOMYOPATHY_HCM	KEGG_HYPERTROPHIC_CARDIOMYOPATHY_HCM	83	-0.321406839	-1.650212484	0.00449715309252392	0.0258586302820126	0.0181957181046198	8280	tags=63%	list=39%	signal=38%	TPM4/DMD/PRKAB1/CACNA2D1/CACNG2/CACNB4/MYH7/ITGA10/ITGA8/CACNA1D/CACNG6/PRKAG2/ITGA3/TPM2/CACNB2/CACNB3/CACNG4/ACTC1/EMD/PRKAA2/CACNA1F/CACNA1C/CACNG3/DAG1/LMNA/SGCG/ITGA11/MYL3/CACNG8/IGF1/TNF/ITGA7/RYR2/TGFB1/CACNG7/MYH6/PRKAG3/MYL2/ITGA9/SGCA/ACTB/ACTG1/CACNB1/CACNA1S/ITGA5/CACNG1/CACNA2D4/TNNT2/TNNI3/MYBPC3/ITGB4/DES
NUP133	KEGG_ARRHYTHMOGENIC_RIGHT_VENTRICULAR_CARDIOMYOPATHY_ARVC	KEGG_ARRHYTHMOGENIC_RIGHT_VENTRICULAR_CARDIOMYOPATHY_ARVC	KEGG_ARRHYTHMOGENIC_RIGHT_VENTRICULAR_CARDIOMYOPATHY_ARVC	74	-0.320738816	-1.610073156	0.00734191771800312	0.0409367533367447	0.0288056101854668	8021	tags=55%	list=38%	signal=35%	CACNG2/CACNB4/ITGA10/ITGA8/CACNA1D/CACNG6/CTNNA3/ITGA3/DSG2/CACNB2/CACNB3/CACNG4/EMD/CACNA1F/CACNA1C/CACNG3/DAG1/LMNA/SGCG/ITGA11/ACTN1/CACNG8/CDH2/ITGA7/RYR2/TCF7L1/CACNG7/ACTN3/CTNNA2/ITGA9/ACTN4/SGCA/ACTB/ACTG1/CACNB1/CACNA1S/ITGA5/CACNG1/CACNA2D4/ITGB4/DES

NUP133	KEGG_PHENYLALANINE_METABOLISM	KEGG_PHENYLALANINE_METABOLISM	KEGG_PHENYLALANINE_METABOLISM	17	-0.521191054	-1.774978596	0.00786106404988011	0.0425422289758218	0.029935321304497	5442	tags=47%	list=26%	signal=35%	DDC/AOC3/ALDH3A1/IL4I1/HPD/ALDH1A3/ALDH3B2/ALDH3B1
NUP133	KEGG_ONE_CARBON_POOL_BY_FOLATE	KEGG_ONE_CARBON_POOL_BY_FOLATE	KEGG_ONE_CARBON_POOL_BY_FOLATE	17	0.515431222795149	1.73697912976957	0.0102808301507558	0.0485044294292069	0.0341306911887744	4134	tags=47%	list=20%	signal=38%	ATIC/MTR/MTFMT/GART/MTHFD2L/MTHFD1L/MTHFD1/DHFR
NUP1	KEGG_ARACHIDONIC_ACID_ME	KEGG_ARACHIDONIC_ACID_ME	KEGG_ARACHIDONIC_ACID_ME	57	-0.348	-1.618	0.0101	0.0485	0.0341	8156	tags=5	list=	signa	PLA2G3/GGT6/PTGIS/PTGS1/ALOX12B/CYP4A22/PLA2G4E/PLA2G2A/PTGDS/PLA2G4B/JMJD7-PLA2G4B/GPX2/CYP4F2/CYP2B6/GPX1/ALOX15/PLA2G2F/PLA2G2D/ALOX15B/ALOX5/PLA2G2E/PTGS

33	TABOLISM	TABOLISM	TABOLISM		699231	757483	3080599322122	0442942920669	30691188744		1%	39%	1=31%	2/GPX3/CYP4F3/PTGES/GGT5/PTGES2/GGT1/LTC4S
NUP133	KEGG_PURINE_METABOLISM	KEGG_PURINE_METABOLISM	KEGG_PURINE_METABOLISM	154	0.258595685781349	1.45263127937638	0.0100258187688926	0.0485044294292069	0.034130691188744	4703	tags=32%	list=22%	signal=25%	POLR2B/POLR1B/GMPS/POLA1/ATIC/PDE7A/PDE8A/PRPS1/ADPRM/ENTPD4/PDE3B/PNPT1/NME7/POLR3B/HPRT1/RRM1/NT5E/POLR3F/GART/AK5/PRIM2/NUDT5/POLR1E/PPAT/PRIM1/POLR3A/RRM2B/POLR1C/IMPDH2/NT5C3A/POLR3C/ENTPD5/ADSL/POLR2K/PAICS/AK2/ADK/PDE7B/PDE4D/DCK/POLR3K/ITPA/POLR2D/NUDT2/ENPP3/PDE4B/NUDT9/ADA/POLR2H
NUP133	KEGG_CHEMOKINE_SIGNALING_PATHWAY	KEGG_CHEMOKINE_SIGNALING_PATHWAY	KEGG_CHEMOKINE_SIGNALING_PATHWAY	187	-0.240508491	-1.40535137	0.010236988865	0.04850442942	0.03413069118	7389	tags=42%	list=35%	signal=27%	CXCL9/SHC4/CCL16/PLCB4/GNB1/GNG3/CXCL5/ADCY5/GNGT1/ADCY2/CXCL3/CCL27/CXCL12/GNG12/CCL17/CCL3L3/JAK3/NFKBIA/PRKCD/CX3CL1/CCR1/CXCR2/ADCY7/CCL3/NCF1/SHC3/HCK/CCL19/CXCL14/GNG4/ADCY6/CXCL8/CXCL16/CCL13/GRK4/CCL24/CCL22/PRKACA/XCR1/CCL21/GRK6/BCAR1/IKBKG/CCL26/AKT1/WASL/PIK3R2/RAC2/CSK/PXN/PIK3R5/FOXO3/MAPK3/PIK3CD/PTK2B/RHOA/GRK2/HRAS/CXCL2/SHC2/PREX1/

							75756	92069	8744				CCR10/GNAI2/GNG8/PLCB2/AKT2/GNG13/ADCY4/SHC1/ARRB2/CXCL1/PLCB3/FGR/GSK3A/RELA/WAS/GNB2/NFKBIB
NUP133	KEGG_MAPK_SIGNALING_PATHWAY	KEGG_MAPK_SIGNALING_PATHWAY	KEGG_MAPK_SIGNALING_PATHWAY	265	-0.22609275	-1.387398701	0.00950086128381264	0.0485044294292069	0.034130691188744	6593	tags=45%	list=31%	signal=31%	TP53/PTPN5/MAP3K13/PLA2G4E/BDNF/MAP4K2/CACNB2/PPP5C/RASGRF1/CACNB3/GNG12/PLA2G2A/MAP3K3/CACNG4/RRAS/PLA2G4B/JMJD7-PLA2G4B/MAPKAPK3/MAPT/TRAF2/PDGFB/CACNA1F/CACNA1C/MAP3K14/CACNG3/CACNA1A/JUN/DUSP3/HSPB1/CHP2/HSPA6/CD14/FGF22/DUSP4/PLA2G2F/FGF12/PLA2G2D/NTRK1/NR4A1/DUSP10/FGF8/CACNG8/IL1B/CACNA1E/TNF/MAPK11/TAB1/TGFB1/MAP2K7/FGF13/FGF16/FGF3/PLA2G2E/CACNG7/FGF18/FGF4/PRKACA/FGFR3/MAPK7/FGF17/FGFR4/NGF/IKBKG/AKT1/CACNA1B/FGF2/FGF21/FGF1/RAC2/JUND/FOS/GADD45B/DUSP9/MAP2K3/HSPA1B/HSPA1A/MAPK3/HSPA2/MAPKAPK2/DUSP1/CACNB1/CACNA1S/MAPK8IP2/FLNA/RAC3/HRAS/GADD45G/FLNC/NFKB2/MAPK12/FGF20/CACNG1/ECSIT/NFATC4/RELB/TAOK2/CACNA1H/DUSP8/AKT2/MKNK2/CACNA2D4/ELK1/FGF19/MAP3K6/MAPK8IP3/RPS6KA1/NTF4/ARRB2/MAP2K2/DUSP7/RPS6KA4/MAP3K11/RASGRP4/TNFRSF1A/FGF11/CHP1/SRF/RELA
NUP133	KEGG_GLYCOSPHINGOLIPID_BIOSYNTHESIS_GLOBO_SERIES	KEGG_GLYCOSPHINGOLIPID_BIOSYNTHESIS_GLOBO_SERIES	KEGG_GLYCOSPHINGOLIPID_BIOSYNTHESIS_GLOBO_SERIES	14	-0.5378871	-1.7289727	0.01070278	0.04923282	0.03464323	5254	tags=50%	list=25%	signal=3	NAGA/FUT9/A4GALT/FUT1/GBGT1/FUT2/ST3GAL2

					83	03	86826959	79404011	70518841				8%	
NUP133	KEGG_N_GLYCAN_BIOSYNTHESIS	KEGG_N_GLYCAN_BIOSYNTHESIS	KEGG_N_GLYCAN_BIOSYNTHESIS	46	0.365254976480139	1.64534520576384	0.0113994581230468	0.0499404832057291	0.0351411866951068	3110	tags=35%	list=15%	signal=30%	ALG6/MAN2A1/MAN1A2/ALG11/STT3B/FUT8/MGAT5/ALG10B/ALG10/ALG9/DPM1/MGAT4A/ALG13/ALG8/ALG5/ALG14
NUP133	KEGG_MELANOMA	KEGG_MELANOMA	KEGG_MELANOMA	71	-0.320742602	-1.592195803	0.01117985096894	0.04994048320572	0.03514118669510	5201	tags=42%	list=25%	signal=32%	CCND1/FGF22/MITF/FGF12/FGF8/IGF1/FGF13/FGF16/FGF3/FGF18/FGF4/CDKN1A/FGF17/AKT1/FGF2/E2F2/FGF21/FGF1/PIK3R2/PIK3R5/MAPK3/PIK3CD/HRAS/FGF20/ARAF/AKT2/FGF19/MAP2K2/BAD/FGF11

							01	91	68					
TRMT11	KEGG_RIBOSOME	KEGG_RIBOSOME	KEGG_RIBOSOME	87	0.610927779702158	2.96069432618437	1e-10	6.13333333333333e-09	3.89473684210526e-09	6281	tags=78%	list=30%	signal=55%	RPL30/RPS27A/RPS18/RPS29/RPS7/RPL31/RPS21/RPL13/RPL21/RPL37/RPLP0/RPL14/RPL7A/RPSA/RPL36AL/RPL5/RPS10/RPS3A/RPL27A/RSL24D1/RPL12/RPL34/RPL6/RPS27/RPLP2/RPL23/RPL23A/RPL24/RPL35A/RPS11/RPL17/RPS6/RPL9/RPL32/RPS4X/RPS15A/RPL36A/RPL37A/RPL3/RPL10A/RPS27L/RPS3/RPL18/RPL15/RPL35/RPS2/RPL22L1/RPS28/RPLP1/RPS13/RPL22/RPS16/RPS19/RPL36/MRPL13/RPS25/RPL38/RPL7/RPS26/RPL13A/RPL10/RPS24/RPL27/UBA52/RPL39/RPS8/RPS17/RPS5
TRMT11	KEGG_NEUROACTIVE_LIGAND_RECEPTOR_INTERACTION	KEGG_NEUROACTIVE_LIGAND_RECEPTOR_INTERACTION	KEGG_NEUROACTIVE_LIGAND_RECEPTOR_INTERACTION	272	-0.421908164	-2.565321353	1e-10	6.13333333333333e-09	3.89473684210526e-09	7838	tags=60%	list=37%	signal=38%	EDNRA/GLRB/TSHB/DRD3/NTSR1/AVPR1B/GLP2R/P2RX7/BRS3/P2RY11/ADCYAP1R1/HTR5A/GRM2/GRM6/SCTR/VIPR1/LHCGR/DRD1/HTR4/LPAR1/HTR1F/GRID2/C3AR1/GRM8/GIPR/HCRTR2/NPFFR1/NMBR/GABRD/FPR3/MC4R/NPY2R/PRLR/GABRB1/OPRM1/TACR2/GRM7/GHRHR/GRIN3A/GLP1R/ADRA1A/SSTR5/CCKAR/GABRB2/NTSR2/HTR2C/CHRNE/CHRNB1/OXTR/SSTR3/CHRM2/GABBR2/PTGER4/SSTR4/ADORA1/P2RX6/MLNR/NPFFR2/CHRNA4/GHSR/MCHR1/BDKRB1/PTGER3/TACR1/GRIN1/ADRB3/GRM5/NPBWR1/GABRQ/EDNRB/CRHR2/CHRNB4/LPAR2/HTR1A/CHRM4/THRB/APLNR/F2/OPRK1/BDKRB2/CHRNA10/P2RY1/GPR50/CCKBR/UTS2R/FPR2/TBXA2R/ADRB1/GRIN2C/GALR1/CHRNA3/CHRND/PTGIR/P2RY6/PRLHR/PRSS3/GH1/GLRA1/GABRA

														1/PTGFR/OPRD1/CSH1/GRIN2A/SSTR1/DRD5/HTR1B/GABBR1/ADORA2B/P2RY4/GRM1/GALR3/ADRA2B/SSTR2/ADRA2A/PTH1R/GH2/HRH3/GRIA2/PRL/CHRNB2/GABRB3/F2RL3/GRIK3/HTR7/MTNR1A/GRIN2D/GRM4/LHB/MTNR1B/GRIK5/CHRNG/KISS1R/P2RX1/TSPO/S1PR2/P2RX3/LTB4R2/LTB4R/ADRA2C/HCRTR1/GALR2/DRD2/CHRM1/LEP/GCGR/GABRG3/CRHR1/ADRA1D/GRID1/C5AR1/CHRNA7/P2RX2/PTGER1/DRD4/P2RY2/S1PR3/HRH2/F2RL1/GPR35/FPR1/GRIN3B/PTAFR/OPRL1
TRMT11	KEGG_SPLICEOSOME	KEGG_SPLICEOSOME	KEGG_SPLICEOSOME	126	0.494960316357307	2.54322459611474	1e-10	6.13333333333333e-09	3.89473684210526e-09	4863	tags=50%	list=23%	signal=39%	MAGOHB/THOC2/THOC1/THOC3/U2SURP/RBMX/SRSF1/PRPF3/PPIH/AQR/PRPF38A/NCBP1/LSM8/SF3B1/SNRNP40/SRSF2/HNRNPA3/SNRPA1/SNRPD3/HNRNPA1L2/PLRG1/HNRNPU/DDX5/DDX46/SRSF10/SNRPF/PRPF40A/SRSF8/LSM6/HNRNPA1/PRPF38B/SRSF6/SRSF3/SRSF5/DHX15/PPIL1/SNW1/HSPA1L/SNRNP200/CDC5L/DDX42/SLU7/LSM3/CDC40/PRPF4/RBM25/PRPF8/LSM5/RBM17/SF3B3/PRPF18/SNRPE/SNRPG/ISY1/HNRNPK/SNRNP27/HNRNPC/HNRNPM/TXNL4A/TCERG1/SNRPD1/DDX39B/SF3A3
TRMT11	KEGG_CELL_CYCLE	KEGG_CELL_CYCLE	KEGG_CELL_CYCLE	123	0.4451777	2.2818895	1.1266653	5.1826066	3.2910014	4633	tags=4	list=2	signal=	ANAPC1/CDC14A/ORC5/RBL1/ATR/ORC2/ATM/SMAD2/STAG1/WEE1/ORC4/MAD2L1/HDAC1/TFDP2/CDK6/PRKDC/STAG2/CCNH/CDC23/BUB3/RBL2/SMC3/ORC3/ANAPC7/RAD21/YWHAZ/SKP1/YWHAQ/MCM6/ANAPC10/CUL1/MCM3/YWHAB/CDC16/CDC25B/ANAPC4/CCNE1/SMAD4

					320005536	33184061	54572209e-08	31032162e-07	30460927e-07		3%	2%	34%	/CDKN1B/SKP2/CCND2/CDC27/CDK2/CCNE2/CHEK1/MCM7/MDM2/DBF4/E2F5/ANAPC5/CDK7/PTTG2/CDC7
TRMT11	KEGG_UBIQUITIN_MEDIATED_PROTEOLYSIS	KEGG_UBIQUITIN_MEDIATED_PROTEOLYSIS	KEGG_UBIQUITIN_MEDIATED_PROTEOLYSIS	129	0.426175164583949	2.20150982594809	1.20504207771775e-07	4.43455484600134e-06	2.81599306582465e-06	4717	tags=43%	list=22%	signal=34%	ANAPC1/WWP1/CUL2/FBXO4/BIRC3/UBE2Q2/BIRC6/HERC4/UBE3A/FBXW7/UBA6/PIAS2/CDC23/CUL3/UBA2/TRIM37/HERC3/RCHY1/CUL4A/CUL5/HERC1/ANAPC7/ERCC8/CBLB/HERC2/SMURF2/BTRC/SKP1/FANCL/DET1/ITCH/UBE2D2/NEDD4/ANAPC10/CUL1/XIAP/UBE2G1/CDC16/ANAPC4/CUL4B/UBR5/KLHL9/UBE2N/BIRC2/RNF7/FBXW11/SKP2/TRAF6/CDC27/MDM2/ANAPC5/UBOX5/UBE2W/UBE2E1/UBE2K/UBA3
TRMT11	KEGG_RNA_DEGRADATION	KEGG_RNA_DEGRADATION	KEGG_RNA_DEGRADATION	52	0.5451724258	2.3549237988	7.2749099684	2.2309723903	1.4166929938	5371	tags=63%	list=25%	signal=47%	DDX6/EXOSC3/ZCCHC7/CNOT6L/DIS3/EXOSC7/CNOT6/EXOSC9/PAPOLG/LSM8/CNOT2/CNOT1/PARN/EDC3/PNPT1/XRN1/LSM6/ENO2/CNOT9/CNOT10/CNOT7/EXOSC2/LSM3/LSM5/EXOSC8/PAPOLA/CNOT8/HSPA9/EXOSC10/CNOT4/DCP1A/EXOSC1/DCP1B

					40979	8942	1462e-07	1382e-05	4916e-05					
TRMT11	KEGG_LYSOSOME	KEGG_LYSOSOME	KEGG_LYSOSOME	120	-0.368560187	-2.002509157	5.59987159111803e-06	0.000147196624680817	9.34715408442257e-05	3859	tags=38%	list=18%	signal=32%	IDUA/NAGLU/SORT1/AP4M1/NAGPA/CTNS/CLTA/CTSA/CTSE/GAA/GGA1/TCIRG1/CD63/ATP6V0A1/PLA2G15/CTSH/CTSD/HEXB/ATP6AP1/ACP2/PSAPL1/NAGA/MAN2B1/TPP1/GNPTG/ARSB/GALNS/HYAL1/CTSK/ATP6V0D1/CLTCL1/CLTB/GM2A/AP3D1/AP1B1/LAPTM5/AP1S1/ATP6V0B/MCOLN1/NEU1/PSAP/LAMP1/ATP6V0C/CD68/SLC11A1/CTSB
TRMT11	KEGG_DNA_REPLICATION	KEGG_DNA_REPLICATION	KEGG_DNA_REPLICATION	36	0.5510472341159	2.194324042124	1.777996466368	0.0004034898505	0.0002562206716	4448	tags=50%	list=21%	signal=40%	SSBP1/RNASEH2B/RFC1/POLA1/RNASEH1/PRIM2/RFC4/PRIM1/RFC5/RPA1/RFC3/MCM6/MCM3/DNA2/RPA3/POLD2/MCM7/POLA2

					55	01	3e-05	63071	96229					
TRMT11	KEGG_NUCLEOTIDE_EXCISION_REPAIR	KEGG_NUCLEOTIDE_EXCISION_REPAIR	KEGG_NUCLEOTIDE_EXCISION_REPAIR	44	0.520092501159572	2.18441568656274	1.97359166036285e-05	0.000403489850563071	0.000256220671696229	473	tags=45%	list=20%	signal=37%	XPA/RFC1/GTF2H3/ERCC5/ERCC6/CCNH/RFC4/GTF2H1/MNAT1/CUL4A/ERCC8/RFC5/RPA1/RFC3/XPC/ERCC4/CUL4B/RPA3/POLD2/GTF2H2
TRMT11	KEGG_CALCIUM_SIGNALING_PATHWAY	KEGG_CALCIUM_SIGNALING_PATHWAY	KEGG_CALCIUM_SIGNALING_PATHWAY	177	-0.317049766	-1.803263851	2.76327177621795e-	0.0005084420068241	0.0003228664917475	7747	tags=56%	list=37%	signal=36%	NTSR1/AVPR1B/PPP3R2/VDAC1/P2RX7/CACNA1F/HTR5A/CALML6/LHCGR/DRD1/HTR4/SLC25A5/ATP2B2/CHP2/SLC8A1/TACR2/MYLK2/PHKG2/ERBB3/PRKCG/ADRA1A/RYR2/GNAQ/ADCY2/ATP2A1/CACNA1D/CCKAR/PLCE1/PRKACG/CAMK2A/HTR2C/OXTR/CHRM2/P2RX6/PHKA2/BDKRB1/PTGER3/TACR1/GRIN1/ADRB3/GRM5/EGFR/EDNRB/CACNA1C/CACNA1H/GNAS/BDKRB2/NOS2/CCKBR/TBXA2R/CACNA1G/ADRB1/GRIN2C/CACNA1A/MYLK3/PTGFR/CAMK2B/GRIN2A/CALML3/DRD5/ADCY1/ADORA2B/GRM1/NOS1/PDE1B/CACNA1E/CACNA1S/PHKG1/HTR7/PTK2B/PRKACA/ADCY7/GRIN2D/ITPKA/BST1/P2RX1/P2RX3/LTB4R2/CHRM1/CACNA1B/SPHK2/

							05	02	71				RYR1/ADRA1D/SLC8A2/CHRNA7/TNNC2/P2RX2/ADCY4/PTGER1/CALML5/GNA11/HRH2/PLCB2/SPHK1/PTAFR/CHP1/GNA15/PLCD3/PLCB3
TRMT11	KEGG_AMINOACYL_TRNA_BIOSYNTHESIS	KEGG_AMINOACYL_TRNA_BIOSYNTHESIS	KEGG_AMINOACYL_TRNA_BIOSYNTHESIS	22	0.637765663872961	2.23543134585963	5.4878679505146e-05	0.000841473085745572	0.000534345037286948	3487	tags=55%	list=16%	signal=46%	RARS2/FARSB/LARS2/NARS2/SEPSECS/FARS2/MTFMT/WARS2/YARS2/IARS2/HARS2/DARS2
TRMT11	KEGG_MATURITY_ONSET_DIABETES_OF_THE_YOUNG	KEGG_MATURITY_ONSET_DIABETES_OF_THE_YOUNG	KEGG_MATURITY_ONSET_DIABETES_OF_THE_YOUNG	25	-0.595446409	-2.24139334	5.0326942245007e-	0.0008414730857455	0.0005343450372869	6996	tags=76%	list=33%	signal=51%	BHLHA15/HNF1B/FOXA2/NEUROG3/NR5A2/FOXA3/NKX2-2/PAX4/HES1/PDX1/GCK/INS/PKLR/NKX6-1/HHEX/MAFA/HNF4A/MNX1/ONECUT1

							05	72	48				
TRMT11	KEGG_VALINE_LEUCINE_AND_ISOLEUCINE_DEGRADATION	KEGG_VALINE_LEUCINE_AND_ISOLEUCINE_DEGRADATION	KEGG_VALINE_LEUCINE_AND_ISOLEUCINE_DEGRADATION	43	0.496448614802097	2.06887861661673	6.49911694920906e-05	0.000919875014349589	0.000584131158997737	4724	tags=51%	list=22%	signal=40%	OXCT1/ACADSB/BCKDHB/HIBCH/DBT/MCCC2/AUH/MCCC1/HMGCS1/ACADM/HIBADH/ACAT1/PCCA/HADH/MCEE/ACAT2/ALDH9A1/DLD/IVD/PCCB/ALDH3A2/ACAD8
TRMT11	KEGG_MISMATCH_REPAIR	KEGG_MISMATCH_REPAIR	KEGG_MISMATCH_REPAIR	23	0.627141561040085	2.24521912604568	7.40923992356311e-05	0.000973785818525437	0.000618365136477823	3836	tags=61%	list=18%	signal=50%	SSBP1/MSH2/PMS2/RFC1/RFC4/RFC5/RPA1/RFC3/MSH3/MSH6/MLH1/MLH3/RPA3/POLD2

TRMT11	KEGG_PROTEIN_EXPORT	KEGG_PROTEIN_EXPORT	KEGG_PROTEIN_EXPORT	23	0.617310936881525	2.21002467115234	0.000112491653679562	0.00137989761846929	0.000876250776030269	3647	tags=57%	list=17%	signal=47%	IMMP2L/SRP9/IMMP1L/SEC62/SEC61A2/SRP54/SRPRB/SPCS3/SRPRA/SEC63/SPCS1/SRP72/SRP68
TRMT11	KEGG_MELANOGENESIS	KEGG_MELANOGENESIS	KEGG_MELANOGENESIS	101	-0.35664004	-1.873795622	0.000131223323223421	0.00150906821706935	0.000958275584065775	6367	tags=51%	list=30%	signal=36%	PRKCG/MAP2K1/KITLG/MAP2K2/GNAQ/ADCY2/FZD4/PRKACG/CAMK2A/WNT3A/ADCY5/CREBBP/WNT2B/EDNRB/GNAS/WNT7B/WNT8A/ADCY6/WNT5B/RAF1/CREB3L1/CREB3L2/TCF7L1/CAMK2B/CALML3/HRAS/ADCY1/CREB3L3/WNT10A/WNT9A/FZD10/PRKACA/ADCY7/WNT6/WNT9B/WNT4/FZD9/DVL1/FZD8/WNT3/MAPK3/WNT5A/WNT11/MITF/GNAI2/FZD5/ADCY4/CALML5/PLCB2/FZD1/FZD2/PLCB3
TR	KEGG_BASAL_C	KEGG_BASAL_C	KEGG_BASAL_C	55	-0	-1	0.	0.	0.	46	ta	l	s	SUFU/WNT7B/SHH/WNT8A/GLI3/WNT5B/BMP2/TCF7L1/GLI2/BMP4/

MT11	ELL_CARCINOMA	ELL_CARCINOMA	ELL_CARCINOMA		.415469393	.930528868	000375767882005762	00406713472288589	00258267708375477	41	gs=49%	ist=22%	ignal=38%	SMO/WNT10A/WNT9A/FZD10/WNT6/WNT9B/WNT4/APC2/FZD9/DVL1/FZD8/WNT3/WNT5A/WNT11/FZD5/FZD1/FZD2
TRMT11	KEGG_LEISHMANIA_INFECTION	KEGG_LEISHMANIA_INFECTION	KEGG_LEISHMANIA_INFECTION	72	-0.380490324	-1.868129914	0.00040307391847074	0.00412031116658978	0.00261644473393287	5121	tags=46%	list=24%	signal=35%	FOS/JUN/HLA-DMB/HLA-DQB1/PTGS2/NOS2/IL1B/HLA-DMA/IL10/FCGR2A/TGFB1/FCGR1A/HLA-DRB5/MAPK12/MAPK11/HLA-DRB1/ITGB2/HLA-DRB4/HLA-DRB3/PTPN6/RELA/CYBA/CR1/NCF1/MAPK3/NFKBIB/NCF2/TLR4/ITGAM/MYD88/IRAK1/IFNGR2/NCF4
TRMT11	KEGG_NOTCH_SIGNALING_PATHWAY	KEGG_NOTCH_SIGNALING_PATHWAY	KEGG_NOTCH_SIGNALING_PATHWAY	47	-0.4314	-1.9316	0.0050	0.0489	0.0310	5254	tags=5	list=2	signal	DTX4/CREBBP/LFNG/RBPJL/JAG2/PSEN2/HES5/HES1/RFNG/NOTCH1/NUMB/CTBP1/NOTCH4/NCSTN/DVL1/MAML3/JAG1/NOTCH2/NCOR2/NUMBL/MAML1/NOTCH3/PTCRA/CTBP2

					02769	65438	523243718787863	277728645088	696955832979		1%	5%	=38%	
TRMT11	KEGG_COMPLEMENT_AND_COAGULATION_CASCADES	KEGG_COMPLEMENT_AND_COAGULATION_CASCADES	KEGG_COMPLEMENT_AND_COAGULATION_CASCADES	69	-0.382575839	-1.874400424	0.000554917318719812	0.00492559269414002	0.00312780771767473	7668	tags=55%	list=36%	signal=35%	FGA/C4B/C4A/MASP1/C1R/C3AR1/CFI/C9/C8B/SERPINC1/SERPINE1/C8G/C1QB/CFD/F10/F5/BDKRB1/VWF/FGB/F3/F2/F7/BDKRB2/PLAU/C1QA/SERPINA5/SERPINF2/PLAT/C1QC/CD55/CR1/F12/C2/THBD/SERPINA1/C5AR1/PLAUR/CFB
TRMT11	KEGG_GAP_JUNCTION	KEGG_GAP_JUNCTION	KEGG_GAP_JUNCTION	87	-0.3480827	-1.7734641	0.00056216	0.00492559	0.00312780	6367	tags=47%	list=30%	signal=3	PRKCG/MAP2K1/MAP2K2/GNAQ/ADCY2/PRKACG/HTR2C/TJP1/TUBAL3/ADCY5/GRM5/EGFR/CSNK1D/GNAS/TUBB2A/ADCY6/ADRB1/RAF1/GRB2/HRAS/ADCY1/GJD2/GRM1/TUBB3/TUBA3E/PRKACA/ADCY7/TUBB6/TUBA3C/MAPK7/MAPK3/TUBA3D/DRD2/TUBB8/GNAI2/ADCY4/GNA11/SRC/PLCB2/TUBA1A/PLCB3

					21	97	0035744241	26941402	771767473				3%	
TRMT11	KEGG_CYTOKINE_CYTOKINE_RECEPTOR_INTERACTION	KEGG_CYTOKINE_CYTOKINE_RECEPTOR_INTERACTION	KEGG_CYTOKINE_CYTOKINE_RECEPTOR_INTERACTION	262	-0.251375847	-1.519547448	0.000808451784640484	0.00676159674426586	0.00429369129641597	5074	tags=29%	list=24%	signal=23%	IL4R/EGFR/IL25/IL1R2/XCR1/CXCL8/LIF/CXCL12/TNFRSF4/TNFRSF18/CD70/CCL19/IL3/CXCL5/TNFSF13B/IL1B/CCL21/TPO/BMP7/IFNL3/BMP2/CCL17/TNFSF9/IL10/IL11/EPO/CXCR2/TGFB1/GH1/CX3CL1/BMPR1B/ACVR1B/CCL24/EPOR/INHBA/TNFRSF10D/GH2/PRL/CSF1R/TNFRSF10C/CXCL16/IL22RA1/CTF1/INHBB/CNTFR/CCL22/TNFSF12/TNFSF13/TNFRSF8/OSM/NGFR/CXCL2/LEP/TNFRSF6B/CCR2/CSF2RA/IL17B/CCR1/CSF3/IL15RA/ACVRL1/TNFRSF1B/IL18/AMH/IL10RB/IFNGR2/IL6R/TNFRSF1A/CSF3R/TNFRSF12A/PLEKHO2/VEGFA/RELT/CSF2RB/IL17RA/CXCL1/LTBR
TRMT11	KEGG_REGULATION_OF_ACTIN_CYTOSKELETON	KEGG_REGULATION_OF_ACTIN_CYTOSKELETON	KEGG_REGULATION_OF_ACTIN_CYTOSKELETON	211	-0.263347541	-1.540259908	0.000877704103	0.007021632828	0.004458817185	5695	tags=37%	list=27%	signal=28%	PAK6/FGF12/MYL2/CHRM2/ARPC3/ARAF/BDKRB1/FGF1/EGFR/FGF4/PAK5/CHRM4/CFL1/F2/ARPC5/RAC1/VAV2/PIK3R2/GIT1/IQGAP3/ITGA7/BDKRB2/FGF18/ITGA9/RAF1/FGF2/MYLK3/CSK/PIK3CD/RHOA/HRAS/FGD1/FGF22/LIMK1/DOCK1/PAK1/BCAR1/VAV1/FGFR4/ARHGEF4/FGF20/PIP5K1C/ITGB2/INS/FGF3/MYL5/FGF8/PXN/LIMK2/INSRR/BAIAP2/PPP1CA/SLC9A1/APC2/ACTB/FGF17/FGF21/MYL7/ITGAX/MAPK3/PFN3/PFN1/CYFIP1/CHRM1/PAK4/FGF11/GSN/I

							536798	29438	01531		TGA5/FGF19/MYH14/FGFR3/WAS/SSH3/ITGB4/RAC3/CD14/WASF1/ACTG1/ITGAM
TRMT11	KEGG_RNA_POLYMERASE	KEGG_RNA_POLYMERASE	KEGG_RNA_POLYMERASE	28	0.51586447544488	1.94751848267057	0.0010157813979449	0.00735083307284223	0.00466786310689638	7110	tags=79%, list=34%, signal=52%	POLR1B/POLR3F/POLR1E/POLR3B/POLR2B/POLR3K/POLR3C/POLR3A/POLR1C/POLR2C/POLR2D/POLR2F/POLR3D/POLR2K/POLR2H/POLR3GL/POLR1A/POLR2G/POLR1D/POLR3G/POLR2J3/POLR2J2
TRMT11	KEGG_N_GLYCAN_BIOSYNTHESIS	KEGG_N_GLYCAN_BIOSYNTHESIS	KEGG_N_GLYCAN_BIOSYNTHESIS	46	0.453355042971094	1.92762107492825	0.00103870467333	0.007350833072842	0.004667863106896	2098	tags=33%, list=10%, signal=29%	FUT8/MGAT4A/ALG9/ALG6/MGAT5/ALG10B/ALG10/ALG11/MAN2A1/MAN1A2/ALG5/DPM1/STT3B/ALG13/ALG8

							64	23	38					
TRMT11	KEGG_CHEMOKINE_SIGNALING_PATHWAY	KEGG_CHEMOKINE_SIGNALING_PATHWAY	KEGG_CHEMOKINE_SIGNALING_PATHWAY	187	-0.269188388	-1.54446444	0.00100497106962631	0.00735083307284223	0.00466786310689638	6567	tags=43%	list=31%	signal=30%	CCL26/CCL3/AKT2/CCL23/MAP2K1/NFKBIA/ADCY2/CCL13/IKBKG/PRKACG/GNG12/SHC4/CXCL3/CCL8/CXCL14/GRK6/CXCL6/ADCY5/GNG5/XCR1/CXCL8/CXCL12/CCL19/FOXO3/DOCK2/RAC1/GNG4/VAV2/GRK1/CXCL5/PIK3R2/CCL21/ADCY6/RAF1/CCL17/GRB2/CXCR2/CX3CL1/CSK/PIK3CD/RHOA/CCL24/HRAS/ADCY1/PAK1/BCAR1/PREX1/VAV1/SHC2/PXN/PTK2B/CXCL16/PRKACA/ADCY7/RELA/AKT1/CCL22/GNG13/NCF1/GRK2/MAPK3/GNG8/NFKBIB/HCK/GSK3A/CXCL2/PRKCD/SHC1/CCR2/CCR1/WAS/GNAI2/ADCY4/ARRB1/PLCB2/ARRB2/FGR/GNB2/CXCL1/PLCB3
TRMT11	KEGG_GLYCOSYLPHOSPHATIDYLINOSITOL_GPI_ANCHOR_BIOSYNTHESIS	KEGG_GLYCOSYLPHOSPHATIDYLINOSITOL_GPI_ANCHOR_BIOSYNTHESIS	KEGG_GLYCOSYLPHOSPHATIDYLINOSITOL_GPI_ANCHOR_BIOSYNTHESIS	25	0.534684496055362	1.94690585819624	0.00153688668711943	0.0100995410867848	0.00641332414549835	4254	tags=52%	list=20%	signal=42%	PIGL/PIGN/PIGW/PIGF/PGAP1/PIGK/PIGB/PIGU/PIGG/PIGX/PIGP/GPLD1/PIGT
TR	KEGG_RETINOL	KEGG_RETINOL	KEGG_RETINOL	64	-0	-1	0.	0.	0.	76	ta	l	s	CYP1A1/ADH1A/CYP1A2/ADH1C/UGT1A3/UGT1A1/UGT1A4/UGT1A9/

MT11	_METABOLISM	_METABOLISM	_METABOLISM		.368487415	.77557418	00153478168706964	0100995410867848	00641332414549835	41	gs=47%	ist=36%	ignal=30%	UGT1A5/UGT1A6/UGT1A7/UGT1A8/UGT1A10/CYP2B6/DHRS4L2/DHRS4/RDH10/CYP2A13/CYP2A7/CYP2A6/RDH5/CYP4A22/BCO1/CYP26A1/RDH8/CYP26C1/ALDH1A2/CYP26B1/DGAT2/DGAT1
TRMT11	KEGG_BASAL_TRANSCRIPTION_FACTORS	KEGG_BASAL_TRANSCRIPTION_FACTORS	KEGG_BASAL_TRANSCRIPTION_FACTORS	35	0.464790687438959	1.84659369154407	0.00169295580568437	0.0107415126981353	0.00682098346391888	4173	tags=43%	list=20%	signal=34%	TAF4B/GTF2H3/GTF2I/TAF2/GTF2H1/GTF2F2/TAF7/GTF2E2/TBPL1/GTF2E1/TAF9B/GTF2A1/TAF11/TAF5/GTF2H2
TRMT11	KEGG_HEDGEHOG_SIGNALING_PATHWAY	KEGG_HEDGEHOG_SIGNALING_PATHWAY	KEGG_HEDGEHOG_SIGNALING_PATHWAY	56	−0.3904	−1.8303	0.0175	0.0178	0.0685	6084	tags=5	list=2	signal=1	GLI1/PRKACG/WNT3A/LRP2/WNT2B/DHH/CSNK1D/SUFU/WNT7B/SHH/WNT8A/GLI3/BMP7/GAS1/WNT5B/BMP2/CSNK1A1L/BMP8B/GLI2/BMP4/SMO/WNT10A/WNT9A/PRKACA

					15519	81737	921369896391	9844020312	167440649102		7%	9%	=41%	/WNT6/IHH/WNT9B/WNT4/WNT3/ZIC2/WNT5A/WNT11
TRMT11	KEGG_AXON_GUIDANCE	KEGG_AXON_GUIDANCE	KEGG_AXON_GUIDANCE	129	-0.28643156	-1.564460877	0.00191553992887349	0.0113696563520233	0.00721986187113606	6908	tags=50%	list=33%	signal=34%	EFNA5/SEMA5A/CHP2/PLXNC1/UNC5C/CDK5/SLIT1/EFNA1/DCC/EFNA3/PAK6/EFNA4/UNC5D/EFNB2/SEMA3B/SEMA3F/EPHB4/CXCL12/PAK5/RGS3/CFL1/RAC1/EFNB3/NGEF/NRP1/EPHB6/ROBO3/SRGAP3/EPHA2/SRGAP2/SEMA5B/SEMA6C/RHOA/PLXNB3/HRAS/LIMK1/EPHB2/NTN3/PAK1/SLIT3/DPYSL5/LIMK2/SEMA4B/SEMA4A/DPYSL2/NFATC4/SEMA3G/SEMA4G/MAPK3/NTN1/PAK4/PLXNB1/EPHA8/UNC5A/PLXNB2/EFNA2/GNAI2/RAC3/PLXNA2/SEMA6B/EFNB1/UNC5B/CHP1/EPHB3/FES
TRMT11	KEGG_T_CELL_RECEPTOR_SIGNALING_PATHWAY	KEGG_T_CELL_RECEPTOR_SIGNALING_PATHWAY	KEGG_T_CELL_RECEPTOR_SIGNALING_PATHWAY	108	0.33169141431	1.66161895219	0.00218914755	0.01258759841	0.00799326901	5304	tags=44%	list=25%	signal=33%	PPP3CB/PIK3CA/MALT1/DLG1/MAPK9/AKT3/NCK1/PPP3CC/ITK/PRKCQ/PIK3R1/MAP3K7/CD3G/FYN/RASGRP1/CD28/CD3D/ICOS/CBLB/PTPRC/PPP3R1/NFATC3/NFAT5/SOS1/LCK/KRAS/CTLA4/CD40LG/PLCG1/NRAS/IFNG/ZAP70/CD8B/GRAP2/NCK2/MAPK13/CARD11/CD247/PPP3CA/CD3E/PIK3CB/PIK3R3/NFATC2/LAT/PDPK1/IKBKB/CD8A/PIK3CG

					9582	884	113711	90384	895459				
TRMT11	KEGG_TYPE_II_DIABETES_MELLITUS	KEGG_TYPE_II_DIABETES_MELLITUS	KEGG_TYPE_II_DIABETES_MELLITUS	47	-0.396170143	-1.773906493	0.00258517379658723	0.0144143023809712	0.00915324693528493	4633	tags=49%, list=22%, signal=38%	CACNA1C/PIK3R2/CACNA1G/CACNA1A/ADIPOQ/PIK3CD/IRS2/PDX1/GCK/CACNA1E/INS/INSR/ABCC8/HK3/PKLR/MAPK3/SLC2A4/CACNA1B/PRKCD/MAFA/SOCS3/HK2/PKM
TRMT11	KEGG_PYRIMIDINE_METABOLISM	KEGG_PYRIMIDINE_METABOLISM	KEGG_PYRIMIDINE_METABOLISM	97	0.332394405828438	1.6416451667035	0.003583050624268	0.0193906269078 0	0.01231326994717	5918	tags=45%, list=28%, signal=33%	NT5E/UPRT/POLR1B/NME7/ENTPD4/POLA1/POLR3F/CTPS2/PRIM2/RRM1/POLR1E/DCK/PRIM1/CTPS1/POLR3B/POLR2B/POLR3K/PNPT1/ENTPD5/AK3/UMPS/POLR3C/POLR3A/NT5C3A/DCTD/RRM2B/POLR1C/POLR2C/POLD2/POLA2/NUDT2/POLR2D/CAD/POLR2F/POLR3D/TYMS/POLR2K/ITPA/POLR2H/CMPK1/DUT/DHODH/POLR3GL/DTYMK

							23	46	57		
TRMT11	KEGG_ECM_RECEPTOR_INTERACTION	KEGG_ECM_RECEPTOR_INTERACTION	KEGG_ECM_RECEPTOR_INTERACTION	84	-0.319288183	-1.618316467	0.00420142580546013	0.0220874956629904	0.0140258124633406	9035	tags=62%, list=43%, signal=36%	COL6A6/LAMA4/SDC4/GP1BA/SV2B/LAMC2/FN1/TNN/ITGA10/THBS2/CD36/ITGB5/LAMC1/IBSP/ITGA11/COL6A1/LAMC3/COL11A1/GP1BB/COL3A1/GP9/LAMA1/CHAD/COL4A6/COL1A2/COL5A2/VWF/LAMB3/ITGA7/RELN/ITGA9/COL11A2/TNC/LAMA5/HSPG2/TNXB/COL1A1/COL5A1/LAMA3/VTN/COL4A1/SDC2/COMP/SDC1/COL2A1/ITGA5/COL4A2/AGRN/COL5A3/ITGB4/LAMB2/SDC3
TRMT11	KEGG_PURINE_METABOLISM	KEGG_PURINE_METABOLISM	KEGG_PURINE_METABOLISM	154	0.286120397453353	1.51950546972264	0.00435179030565229	0.0222424837844451	0.0141242316937838	5314	tags=36%, list=25%, signal=27%	PDE7A/GMPS/NT5E/PRPS1/PDE3B/POLR1B/NME7/ENTPD4/ATIC/POLA1/POLR3F/PRIM2/RRM1/POLR1E/PAICS/DCK/PRIM1/AK5/POLR3B/POLR2B/PPAT/POLR3K/PNPT1/ENTPD5/ADPRM/NUDT5/PDE4B/ENPP3/POLR3C/GART/IMPDH2/PDE4D/POLR3A/NT5C3A/ADK/RRM2B/POLR1C/POLR2C/POLD2/PDE8A/ADSL/PDE9A/ADA/AK2/POLA2/NPR2/NUDT9/AK1/NUDT2/HPRT1/POLR2D/POLR2F/POLR3D/POLR2K/ITPA/PDE7B

TRMT11	KEGG_GNRH_SIGNALING_PATHWAY	KEGG_GNRH_SIGNALING_PATHWAY	KEGG_GNRH_SIGNALING_PATHWAY	100	-0.298772554	-1.566058969	0.00462943520607827	0.0230220561599568	0.0146192690718261	6479	tags=48%	list=31%	signal=33%	ELK1/MMP2/MAP2K1/MAP2K2/GNAQ/ADCY2/CACNA1D/PRKACG/CAMK2A/PLA2G4A/PLA2G2D/ADCY5/EGFR/JUN/CACNA1C/GNAS/PLA2G2E/GNRH2/ADCY6/RAF1/MAP3K3/GRB2/HBEGF/CAMK2B/CALML3/HRAS/ADCY1/MAP2K3/MAPK12/PLD1/MAPK11/MAP2K7/CACNA1S/PTK2B/PRKACA/ADCY7/LHB/MAPK7/PLD2/MAPK3/PRKCD/ADCY4/CALML5/GNA11/SRC/PLCB2/MMP14/PLCB3
TRMT11	KEGG_MAPK_SIGNALING_PATHWAY	KEGG_MAPK_SIGNALING_PATHWAY	KEGG_MAPK_SIGNALING_PATHWAY	265	-0.234471629	-1.425926002	0.00478986134440389	0.0231930128255346	0.014727828510494	6491	tags=43%	list=31%	signal=30%	CACNA2D3/ELK1/AKT2/PRKCG/MAP2K1/HSPB1/MAP2K2/FGF13/CACNA1D/MKNK2/MAPT/NTRK1/CACNG5/IKBKG/PRKACG/GNG12/CACNG7/CACNG8/FGF12/DUSP8/PLA2G4A/CACNB4/PLA2G2D/FGF1/FOS/EGFR/HSPA2/FGF4/JUN/MAPK8IP3/IL1R2/CACNA2D4/NTF4/HSPA6/CACNA1C/RASGRF1/CACNA1H/RAC1/STK3/PTPN5/CACNG6/IL1B/PLA2G2E/FGF18/CACNA1G/RAF1/FGF2/MAPKAPK2/CACNA1A/MAP3K3/MAP3K8/GRB2/TGFB1/TAOK2/FLNA/CACNG1/DUSP1/NGF/HRAS/MAP2K3/FGF22/MAPK12/CACNG4/PAK1/GADD45A/MAPK11/SRF/MAP2K7/DUSP9/DUSP6/FGFR4/CACNA1E/CACNA1S/FGF20/FLNC/ECSIT/FGF3/FGF8/MKNK1/PRKACA/MAPK8IP2/DUSP10/NFKB2/NFATC4/RELA/AKT1/MAPK7/GADD45B/MAPKAPK3/FGF17/FGF21/MAPK3/DUSP7/GADD45G/RELB/FGF11/CACNA1B/FGF19/RPS6KA4/HSPA1B/HSPA1A/RPS6KA1/FGFR3/RAC3/ARRB1/MAP

														3K11/CD14/RASGRP4/ARRB2/DUSP3/TNFRSF1A/CHP1/MAP3K6
TRMT11	KEGG_NON_HOMOLOGOUS_END_JOINING	KEGG_NON_HOMOLOGOUS_END_JOINING	KEGG_NON_HOMOLOGOUS_END_JOINING	12	0.628875202175314	1.8175715252452	0.00512739185181475	0.0235860025183478	0.0149773814618799	4235	tags=42%	list=20%	signal=33%	RAD50/PRKDC/XRCC5/NHEJ1/DCLRE1C
TRMT11	KEGG_CARDIAC_MUSCLE_CONTRACTION	KEGG_CARDIAC_MUSCLE_CONTRACTION	KEGG_CARDIAC_MUSCLE_CONTRACTION	73	-0.329143703	-1.622327146	0.00506932020195768	0.0235860025183478	0.0149773814618799	6776	tags=48%	list=32%	signal=33%	TPM3/ATP1B4/SLC8A1/CACNA2D3/TPM4/FXYD2/RYR2/CACNA1D/CACNG5/CACNG7/CACNG8/ACTC1/COX8A/MYL2/CACNB4/MYL3/CYC1/CACNA2D4/TNNT2/CACNA1C/CACNG6/MYH6/UQCR11/TNNI3/CACNG1/CACNG4/CACNA1S/COX4I2/SLC9A1/COX7A1/ATP1B2/COX6B2/ATP1A2/UQCRC1/COX6A2

TRMT11	KEGG_OOCYTE_MEIOSIS	KEGG_OOCYTE_MEIOSIS	KEGG_OOCYTE_MEIOSIS	112	0.312791434796378	1.57025942465281	0.00567576130025167	0.02547170924991	0.0161748268120138	4884	tags=37%	list=23%	signal=28%	ANAPC1/PPP3CB/PRKACB/PPP3CC/RPS6KA3/CALM1/PPP2R1B/MAD2L1/CDC23/PPP2R5C/SMC3/ANAPC7/ITPR2/YWHAZ/BTRC/SKP1/PPP1CC/YWHAQ/PPP3R1/ANAPC10/CUL1/SGO1/PPP1CB/PPP2R5A/YWHAB/CDC16/ANAPC4/FBXO5/CAMK2D/CCNE1/PPP2CA/FBXW11/CDC27/ITPR3/CDK2/CCNE2/PPP3CA/ANAPC5/PTTG2/ITPR1/PRKX
TRMT11	KEGG_PROPANOATE_METABOLISM	KEGG_PROPANOATE_METABOLISM	KEGG_PROPANOATE_METABOLISM	31	0.462400125006096	1.77270045503353	0.00611294524271947	0.0266626721505597	0.0169311018805042	4720	tags=42%	list=22%	signal=33%	HIBCH/SUCLA2/ACADM/SUCLG2/ACAT1/PCCA/LDHB/ACACA/MCEE/ACAT2/ALDH9A1/PCCB/ALDH3A2
TRMT	KEGG_FC_GAMMA_R_MEDIATED	KEGG_FC_GAMMA_R_MEDIATED	KEGG_FC_GAMMA_R_MEDIATED	96	−0.2	−1.5	0.00	0.02	0.01	8225	tags	lis	sig	PLA2G4E/AMPH/MARCKSL1/MARCKS/CRK/PRKCE/WASL/ARPC1A/PIK3R5/MYO10/RPS6KB2/WASF2/ASAP3/AKT2/PRKCG/MAP2K1/PLA2G4

11	_PHAGOCYTOSIS	_PHAGOCYTOSIS	_PHAGOCYTOSIS		99730025	63074688	626009450046591	66626721505597	69311018805042		=57%	t=39%	nal=35%	A/PLA2G4D/ARPC3/DNM2/CFL1/DNM1/DOCK2/ARPC5/RAC1/VAV2/PIK3R2/PLPP3/RAF1/PLA2G4F/FCGR2A/PIK3CD/FCGR1A/LIMK1/PLD1/PAK1/VAV1/PIP5K1C/LIMK2/SYK/PLPP2/AKT1/NCF1/ASAP1/PLD2/MAPK3/HCK/GSN/PRKCD/SPHK2/WAS/WASF1/SPHK1/GAB2/VASP
TRMT11	KEGG_OLFACTORY_TRANSDUCTION	KEGG_OLFACTORY_TRANSDUCTION	KEGG_OLFACTORY_TRANSDUCTION	387	−0.205861047	−1.304057143	0.0063758563838295	0.0266626721505597	0.0169311018805042	11478	tags=58%	list=54%	signal=27%	OR4D5/OR2B2/OR6C68/OR10K1/OR4S1/OR10H4/OR52N1/OR1L6/OR2T2/OR5T2/OR7C2/OR8K5/OR9K2/OR4C15/OR52E2/OR5F1/OR5K1/OR10G2/OR5M8/OR52N2/OR11H4/OR13C4/OR2B6/OR4K15/OR5AN1/OR11H6/OR2A5/OR4X2/OR2L13/OR6C3/OR2M7/OR6B2/OR5J2/OR12D2/OR4K5/OR9G4/OR7G3/OR2T3/OR2T34/OR2D2/OR5M10/OR5AK2/OR3A1/OR6N1/OR2T4/OR2A2/OR8A1/OR6C75/OR52J3/OR2AT4/OR11A1/CLCA4/OR6C76/OR10A7/OR6B1/OR10H3/OR10S1/OR4N4/OR4B1/OR52R1/OR56A4/OR4K13/OR1J4/OR13J1/OR13C9/OR1N1/OR4A16/OR2A14/GNAL/OR8D4/OR1C1/OR7A10/OR51B5/OR52D1/OR5AC2/OR10Z1/OR6B3/OR51I1/OR52B4/OR5L1/OR4D6/OR8S1/OR2C1/OR1B1/OR1A1/OR56A5/OR4K14/OR56A3/OR5AU1/OR4C13/OR4D10/OR51F2/OR2F1/CALM3/OR5D13/CLCA2/OR2W1/OR52L1/CNGA4/OR5B17/OR6C74/OR5B12/OR4D2/OR13C8/OR7G1/OR10G3/OR4N5/OR2T1/OR4Q3/OR5AP2/OR1K1/OR13H1/OR6A2/OR51G2/OR7A5/OR2G2/OR5M9/OR8J1/OR5I1/OR10P1/OR10H5/OR52M1/OR6V1/OR9A4/GUCA1C/

														OR5D16/OR10T2/OR6K2/OR10J1/OR9A2/OR51Q1/OR2Z1/OR6T1/OR10A4/OR4D11/OR5A2/OR6K6/CNGA3/OR3A3/OR5A1/OR6Y1/OR52N5/GUCA1B/OR51I2/OR4M2/OR10G8/OR52I1/OR10A5/OR51G1/OR56A1/OR8B8/GRK3/OR10G7/OR5C1/OR5H2/OR1E1/OR51D1/OR1G1/CALML6/OR9Q1/OR1A2/OR51E2/OR10H1/OR7D2/OR52W1/OR1D5/OR2J3/OR5L2/OR13C2/OR2T8/OR4K17/OR10J3/OR10AD1/OR2T33/OR2T12/OR10R2/OR1I1/OR2B3/OR5M3/OR56B4/OR10J5/OR52B2/OR10H2/OR4F17/OR4F5/OR4F4/OR7D4/OR1M1/OR13C5/OR10A2/PRKACG/CAMK2A/OR1L3/OR2C3/OR2H2/OR10C1/OR4A5/OR10V1/OR10G9/OR2H1/OR2J2/OR1E2/OR10Q1/OR2S2/OR52K1/OR9Q2/OR2W3/CAMK2B/OR2B11/CALML3/OR2Y1/OR52E8/OR2V2/OR10G4/GUCA1A/OR11L1/PRKACA/OR51E1/CNGB1/OR4D9/OR52K2/GUCY2D/CALML5/ARRB2
TRMT11	KEGG_PATHWAYS_IN_CANCER	KEGG_PATHWAYS_IN_CANCER	KEGG_PATHWAYS_IN_CANCER	322	–0.210870992	–1.304785813	0.00837173233701961	0.0342310833335913	0.0217371295768228	6375	tags=39%	list=30%	signal=28%	AKT2/PRKCG/LAMA1/MAP2K1/KITLG/NFKBIA/RUNX1T1/MAP2K2/CCND1/DCC/FGF13/GLI1/NTRK1/FZD4/AR/IKBKG/PAX8/COL4A6/WNT3A/FGF12/ARAF/CREBBP/BIRC5/FGF1/WNT2B/FOS/PML/EGFR/PGF/FGF4/JUN/CXCL8/SUFU/RAC1/WNT7B/PTGS2/LAMB3/PIK3R2/NCOA4/DAPK3/NOS2/SHH/WNT8A/FGF18/GLI3/WNT5B/BMP2/CBLC/RAF1/FGF2/LAMA5/GRB2/TGFB1/RUNX1/TCF7L1/PIK3CD/DAPK1/RHOA/HRAS/GLI2/FGF22/PLD1/EGLN1/BMP4/PPARG/PIAS3/CASP9/SMO/WNT10A/CTNNA1/NKX3-1/KLK3/WNT9A/FGF20/LAMA3/CSF1R/FGF3/FGF8/MMP9/FZD10/COL4A1/CTBP1/WNT6/WNT9B/NFKB2/BID/WNT4/RELA/APC2/AKT1/FZD9/DVL1/FZD8/WNT3/FGF17/FGF

												21/MAPK3/EPAS1/WNT5A/BAD/FGF11/WNT11/FGF19/COL4A2/CSF2RA/E2F2/CDKN2B/MITF/FGFR3/CDKN1A/FZD5/CTNNA2/GSTP1/RAC3/BAX/LAMB2/FZD1/SPI1/RXRA/CSF3R/VEGFA/RARA/CEBPA/CTBP2/FZD2
TRMT11	KEGG_BUTANOATE_METABOLISM	KEGG_BUTANOATE_METABOLISM	KEGG_BUTANOATE_METABOLISM	33	0.431815653240239	1.67897592176225	0.0108913427415932	0.0435653709663728	0.0276645090232688	3765	tags=39%	list=18%	signal=32%	OXCT1/ALDH5A1/PDHB/HMGCS1/ACAT1/BDH1/L2HGDH/ACSM3/PDHA1/HADH/BDH2/ACAT2/ALDH9A1
TRMT11	KEGG_DILATED_CARDIOMYOPATHY	KEGG_DILATED_CARDIOMYOPATHY	KEGG_DILATED_CARDIOMYOPATHY	90	-0.299312859	-1.539502557	0.0121757714721313	0.0476668500185566	0.0302689951490834	6776	tags=48%	list=32%	signal=33%	TPM3/SLC8A1/CACNA2D3/TPM4/RYR2/ADCY2/CACNA1D/CACNG5/PRKACG/CACNG7/CACNG8/ACTC1/MYL2/SGCG/CACNB4/MYL3/ADCY5/CACNA2D4/TNNT2/CACNA1C/GNAS/ITGA7/CACNG6/MYH6/ADCY6/ITGA9/ADRB1/TNNI3/TGFB1/CACNG1/ADCY1/CACNG4/SGCA/CACNA1S/PRKACA/ADCY7/DES/ACTB/ITGA5/ADCY4/ITGB4/ACTG1/MYBPC3
